# Genetic and Non-Genetic Mechanisms of Resistance to BCR Signaling Inhibitors in B Cell Malignancies

**DOI:** 10.3389/fonc.2020.591577

**Published:** 2020-10-26

**Authors:** Laura Ondrisova, Marek Mraz

**Affiliations:** ^1^Molecular Medicine, CEITEC Masaryk University, Brno, Czechia; ^2^Department of Internal Medicine, Hematology and Oncology, University Hospital Brno and Faculty of Medicine, Masaryk University, Brno, Czechia

**Keywords:** B cell malignancies, ibrutinib, resistance, adaptation, targeted therapy, B cell receptor, BCR inhibitor

## Abstract

The approval of BTK and PI3K inhibitors (ibrutinib, idelalisib) represents a revolution in the therapy of B cell malignancies such as chronic lymphocytic leukemia (CLL), mantle-cell lymphoma (MCL), diffuse large B cell lymphoma (DLBCL), follicular lymphoma (FL), or Waldenström’s macroglobulinemia (WM). However, these “BCR inhibitors” function by interfering with B cell pathophysiology in a more complex way than anticipated, and resistance develops through multiple mechanisms. In ibrutinib treated patients, the most commonly described resistance-mechanism is a mutation in *BTK* itself, which prevents the covalent binding of ibrutinib, or a mutation in *PLCG2*, which acts to bypass the dependency on BTK at the BCR signalosome. However, additional genetic aberrations leading to resistance are being described (such as mutations in the *CARD11*, *CCND1*, *BIRC3*, *TRAF2*, *TRAF3*, *TNFAIP3*, loss of chromosomal region 6q or 8p, a gain of Toll-like receptor (TLR)/MYD88 signaling or gain of 2p chromosomal region). Furthermore, relative resistance to BTK inhibitors can be caused by non-genetic adaptive mechanisms leading to compensatory pro-survival pathway activation. For instance, PI3K/mTOR/Akt, NFkB and MAPK activation, BCL2, MYC, and XPO1 upregulation or PTEN downregulation lead to B cell survival despite BTK inhibition. Resistance could also arise from activating microenvironmental pathways such as chemokine or integrin signaling *via* CXCR4 or VLA4 upregulation, respectively. Defining these compensatory pro-survival mechanisms can help to develop novel therapeutic combinations of BTK inhibitors with other inhibitors (such as BH3-mimetic venetoclax, XPO1 inhibitor selinexor, mTOR, or MEK inhibitors). The mechanisms of resistance to PI3K inhibitors remain relatively unclear, but some studies point to MAPK signaling upregulation *via* both genetic and non-genetic changes, which could be co-targeted therapeutically. Alternatively, drugs mimicking the BTK/PI3K inhibition effect can be used to prevent adhesion and/or malignant B cell migration (chemokine and integrin inhibitors) or to block the pro-proliferative T cell signals in the microenvironment (such as IL4/STAT signaling inhibitors). Here we review the genetic and non-genetic mechanisms of resistance and adaptation to the first generation of BTK and PI3K inhibitors (ibrutinib and idelalisib, respectively), and discuss possible combinatorial therapeutic strategies to overcome resistance or to increase clinical efficacy.

## Introduction

The BCR signaling pathway plays a central role in the onset and progression of mature B cell malignancies, such as chronic lymphocytic leukemia (CLL), mantle-cell lymphoma (MCL), diffuse large B cell lymphoma (DLBCL), follicular lymphoma (FL), or Waldenström’s macroglobulinemia (WM). Activating mutations in the BCR signaling pathway are commonly found in DLBCL, FL, or WM (1). Though these mutations are usually missing in CLL and MCL, BCR signaling is constitutively activated and is a key player in their pathogenesis (2–5). Introducing “B cell receptor (BCR) inhibitors” in recent years has marked a revolution in treating B cell malignancies since many patients are responsive to the inhibitors of BCR-associated kinases BTK or PI3K, such as ibrutinib and idelalisib, respectively. They are now widely used as a first-line treatment or to treat relapsed/refractory diseases. However, the patient response to them varies across B cell malignancies in clinical trials as well as in real-world setting, and a large percentage of patients develop resistance or have to stop the therapy due to toxicities associated with these inhibitors’ long-term use (6–14).

In this review, we summarize the genetic and non-genetic mechanisms of resistance and adaptation to the first generation of BTK and PI3K inhibitors (ibrutinib and idelalisib, respectively), and discuss possible therapeutic strategies to overcome resistance or increase clinical efficacy by using combinatorial therapeutic strategies. We also discuss the complexity of the mechanisms of action of “BCR inhibitors” and how this affects the choice of potential combinatorial therapy.

## BCR Signaling and Its Cross-Talk With Other Pathways

**Figure 1:**
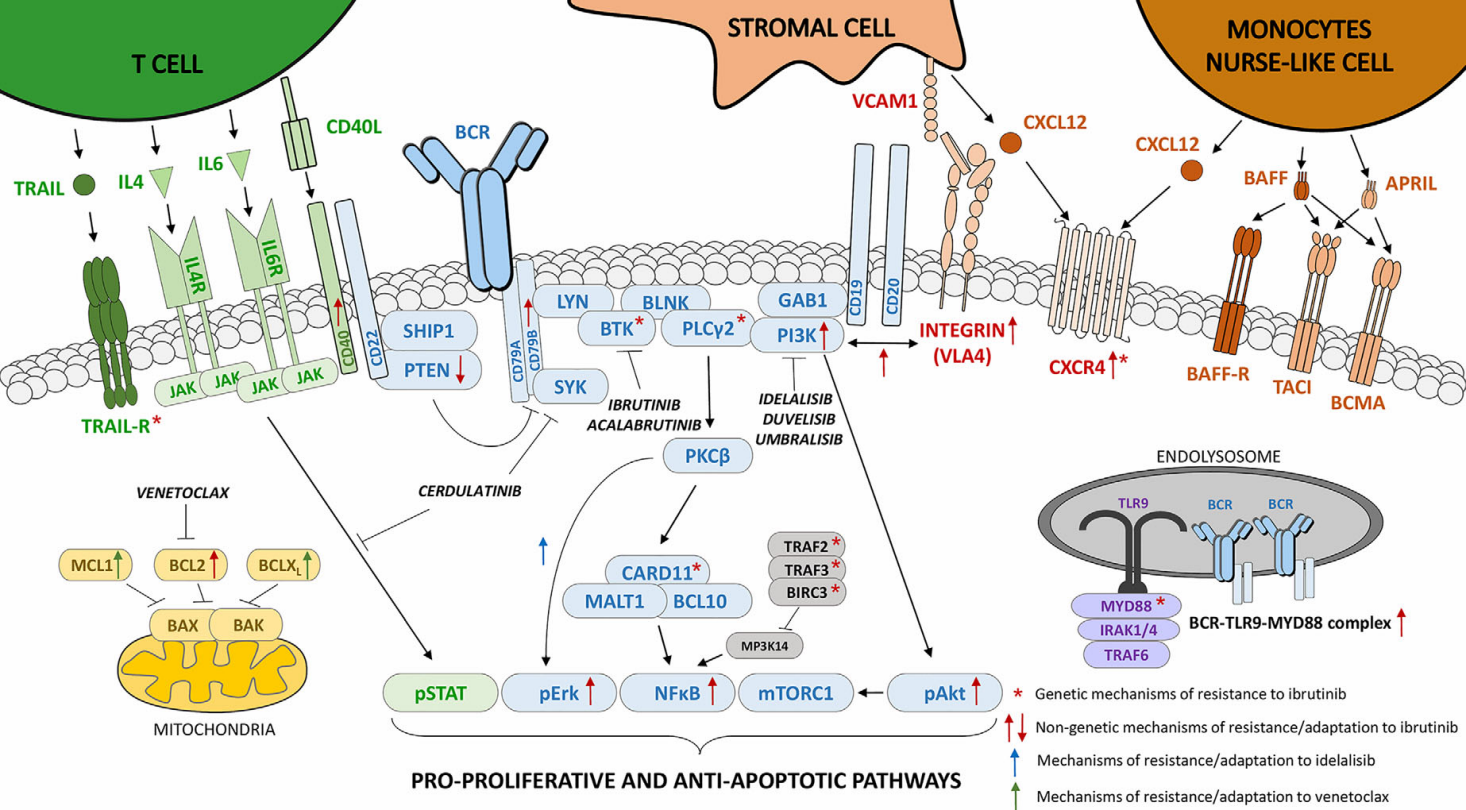
The genetic and non-genetic mechanisms of resistance to BTK or PI3K inhibition in B cell malignancies. The “*” indicates genetic mechanisms of resistance to ibrutinib (BTK inhibitor), the red arrows indicate non-genetic mechanisms of resistance/adaptation to ibrutinib, the blue arrows indicate mechanisms of resistance/adaptation to idelalisib (PI3K inhibitor), and the green arrows indicate mechanisms of resistance/adaptation to venetoclax (BH3-mimetic [BCL2 inhibition]).

Cell-surface immunoglobulin does not have any kinase activity itself. It is non-covalently connected to disulphide-linked heterodimers Igα and Igβ (CD79A, CD79B). After recognition and antigen binding, BCRs start to aggregate and change their conformation, which concludes in phosphorylation of tyrosine-based activation motifs (ITAMs) on Igα and Igβ’s cytoplasmic domains. This phosphorylation, mediated by Src-family kinase LYN, creates a docking site for spleen tyrosine kinase (SYK) (15). The activated SYK then phosphorylates the B cell linker protein (BLNK), an adaptor protein helpful in recruiting other molecules such as Bruton tyrosine kinase (BTK). BCR stimulation also leads to phosphorylation of co-receptor CD19 and PI3K adaptor BCAP by LYN and SYK, which afterwards activates phosphoinositide 3-kinase (PI3K) leading to PIP3 generation (16, 17) (**Figure 1**).

PIP3 helps to recruit GRB2-associated-binding protein 1 (GAB1), 3-phosphoinositide-dependent protein kinase 1 (PDK1, also known as PDPK1), protein kinase B (PKB, Akt), and BTK to the plasma membrane *via* their pleckstrin homology (PH) domain. Here, Akt is phosphorylated on S473 by mTORC2 which also facilitates Akt phosphorylation on T308 by PDK1 leading to full Akt activation (18). PI3K signaling is further positively regulated by the adaptor protein GAB1, which recruits additional PI3K molecules generating more PIP3 (19, 20). On the other hand, the amount of PIP3 is negatively balanced by the activity of phosphatases such as SHIP1, SHP1, and PTEN.

PIP3 is also needed for optimal BTK activation, since it helps to translocate BTK to the cell membrane and *via* the interaction with its PH domain, it allows the activation of BTK’s kinase activity (21). For full BTK activation after the recruitment to the cell membrane, phosphorylation at two sites is needed. Firstly, BTK gets phosphorylated by SYK or LYN at tyrosine Y551, which then leads to autophosphorylation at Y223 (22, 23). Fully activated BTK phosphorylates phospholipase Cγ2 (PLCγ2). PLCγ2 hydrolyses PIP2 into secondary messengers inositol triphosphate (IP3), which controls intracellular Ca^2+^ levels, and diacylglycerol (DAG) which, *via* protein kinase Cβ (PKCβ) activation, induces cRaf-MEK-Erk pathway activation. PKCβ also activates CARD11, which then forms a complex with MALT1 and BCL10 to activate TAK1 (24). Afterwards, TAK1 phosphorylates IKKβ which initiates the NFκB pathway (25). Apart from this, PKCβ plays a role in negative feedback regulation of BCR signaling by removing BTK from the plasma membrane by phosphorylating BTK on S180 (26). Non-redundant negative regulation is also mediated by LYN kinase, since mouse B cells with LYN knockout have a surprisingly stronger BCR signaling suggesting that LYN has a specific role in negatively regulating the pathway (27). BCR signaling propensity is also affected by levels of cell-surface molecules that act as docking sites for positive or negative BCR pathway regulators, which include molecules such as CD19, CD22, and CD32. Recently, we have shown that a notorious therapeutic target in B cell malignancies, CD20, is also a positive BCR signaling regulator (28). When CD20 is silenced, response to BCR stimulation is weaker, as underscored by the lower phosphorylation of BCR-associated kinases and impaired calcium flux (29, 30). Moreover, an additional layer of regulation involves small non-coding RNAs (microRNAs) that influence both the positive and negative regulation of BCR signaling propensity (20, 31–38).

BCR signaling is activated in the lymphatic tissue microenvironment and is closely intertwined with the pathways responsible for the cell homing and adhesion (5). BCR activation affects adhesion *via* integrin VLA4 formed by CD49d and CD29 (integrin β1); together BCR and VLA4 provide B lymphocytes with adhesion and enhanced signaling (39). CD49d activation causes SYK phosphorylation and, on the other hand, BCR stimulation leads to VLA4 activation (40–42). BCR stimulation also increases chemotaxis towards chemokines such as CXCL12 produced in the microenvironment. Binding of CXCL12 to its receptor CXCR4 activates PI3K, MAPK, and STAT3, and leads to actin polymerization and cell migration (43–45). In CLL, cell-surface IgM levels and BCR signaling is increased by the IL4 produced by T cells which also activates the JAK1-3/STAT6 pathway and upregulates the levels of anti-apoptotic proteins from BCL2 family, resulting in partial malignant B cell protection from the effects of “BCR inhibitors” (46, 47). The importance of the microenvironment can be well illustrated in CLL, where malignant B cells are dependent on constant re-circulation between the peripheral blood and lymph nodes, where they are supported by pro-survival signals from mesenchymal stromal cells, monocyte-derived nurse-like cells, and T lymphocytes (29, 43, 48–50). The supportive stromal cells produce not only chemoattractants CXCL12 and CXCL13 but also BAFF, APRIL, CD31, and plexin B1 which protect CLL cells from spontaneous and induced apoptosis by activating BCR and NFκB signaling (43, 49, 51, 52). Kinases of the BCR pathway BTK and PI3Kδ together with JAK are also involved in T cell dependent proliferation induced by CD40L and IL21, which can be inhibited by ibrutinib, idelalisib or JAK inhibitor (53).

Overall, there is crosstalk between the BCR, chemokine signaling and cell adhesion pathways. Therefore, the success of “BCR inhibitors” lies not only in inhibiting the BCR pathway itself but also in inhibiting other processes. In CLL and some lymphomas, BTK/PI3K inhibition results in malignant B cells egressing from the lymph nodes, causing transient lymphocytosis in patients (8, 54, 55).

## Mechanistic Effects of Ibrutinib Action

Ibrutinib is an orally administered small-molecule inhibitor targeting BTK. It binds to BTK covalently, selectively, and irreversibly, inhibiting its phosphorylation and enzymatic activity. BTK is an important kinase in BCR signaling needed for B cells to properly develop (56, 57). Inhibiting it with ibrutinib leads to a loss of pro-survival signals from BCR activation by ligands, and also impairs the “tonic” BCR signals that sustain B cell survival. BTK inhibition decreases cell proliferation as well as interferes with the activation of downstream molecules in BCR pathway such as PLCγ2, Akt and Erk irrespective of BCR stimulation (58–61). As BTK is not only involved in BCR signaling (see above), ibrutinib also disrupts CXCR4 internalization, impairs migration toward CXCL12 and also indirectly decreases total BTK levels (62). Ibrutinib further disrupts signaling from CXCR5 and integrins, molecules that allow B lymphocyte migration and adhesion (63, 64). Altogether, ibrutinib inhibits BCR stimulation, B cell proliferation, and migration toward homing chemokines such as CXCL12 and CXCL13. It also blocks BCR-dependent CCL3 and CCL4 chemokine release in CLL and decreases CCL4, CCL22, and CXCL13 levels in the serum of ibrutinib-treated MCL patients (54, 61). As mentioned above, inhibiting the adhesion and homing capacity causes transient lymphocytosis in CLL and MCL patients (54, 55). In most of the patients, this resolves within 8 months after starting therapy (55). CLL cells together with non-malignant immune cells after ibrutinib treatment are of a quiescent phenotype as shown by the expression of the genes involved in senescence and/or cell quiescence (30, 55, 65). Apart from the mentioned mechanisms of ibrutinib action, it also affects the microRNAs’ expression, resulting in higher levels of several tumor suppressors and inhibition of cell proliferation (66, 67).

Ibrutinib has been approved for therapy of CLL, MCL, WM, and marginal zone lymphoma (MZL). Though it is a potent drug, not all patients are responsive to ibrutinib and a significant number of them acquire resistance to the treatment or discontinue the therapy due to toxicities that are most likely caused by ibrutinib off-target inhibition of molecules such as BLK, JAK3, EGFR, and several TFK members (for a list of off-targets of ibrutinib and other BTK inhibitors see **Supplementary Table 1**). In the following sections, we will summarize the genetic mechanisms of ibrutinib resistance, the non-genetic mechanisms of adaptation/resistance by activating compensatory pro-survival pathways and describe possible solutions to different types of ibrutinib resistance (**Figure 1**).

## Genetic Mechanisms of Ibrutinib Resistance

Genetic mechanisms of primary or acquired resistance to ibrutinib have been widely studied and recurrent mutations associated with resistance have been described in B cell malignancies (**Table 1**, **Figure 1**). Whole-exome sequencing revealed mutations in BCR-involved proteins BTK and PLCγ2 in ~80% of CLL patients with acquired resistance to ibrutinib (7, 96), however, some studies have reported a much lower frequency of these mutations (97, 98). The most common mutation in *BTK* is a C481S point mutation which interferes with the binding of ibrutinib to BTK (7, 68). Other mutations in the *BTK* gene were also found in ibrutinib-resistant patients and have been suggested to affect either ibrutinib binding to BTK or BLNK binding to BTK, altogether leading to PLCγ2 activation even in the presence of ibrutinib (93, 99–101) (**Supplementary Table 2**). *PLCG2*, a gene coding for PLCγ2, seems to mostly harbor gain-of-function mutations when PLCγ2 can be activated by RAC2 or SYK and LYN without BTK (7, 78, 79, 100) (**Supplementary Table 2**). As mutations in *BTK* and *PLCG2* occur early before relapse on ibrutinib, they may serve as a biomarker to indicate a need to change the therapy before disease progression (102). They can also co-occur, which may bring complications to solving the resistance with next-generation BTK inhibitors (103). Apart from the mentioned mutations, resistance to ibrutinib in CLL has also been associated with chromosomal aberrations such as 8p deletion and gain in the 2p region (92, 93). The 8p region contains a gene for TRAIL receptor and its deletion results in insensitivity to TRAIL-induced cell death (93). On the other hand, 2p gain causes exportin-1 overexpression (XPO1), regulating the transport of proteins between the nucleus and cytoplasm (92). Additionally, CLL patients that progress or develop Richter’s transformation on ibrutinib recurrently harbor mutations of tumor-suppressor *TP53*, splicing factor *SF3B1*, or NFκB pathway regulator *CARD11*, however, whether these genetic aberrations may directly impact response to ibrutinib during Richter’s transformation remains unclear (80). Though, despite good initial response in most of CLL patients, it has been shown that mutations in *TP53* are responsible for a worse prognosis in a long-term ibrutinib treatment and they also partially protect CLL cells *in vitro* from ibrutinib-induced apoptosis and inhibition of proliferation (104–107). This might be related to the recently described role of p53 in the negative regulation of BCR signalling (31, 32).

**Table 1:** Recurrent mutations in ibrutinib-resistant patients and possible therapeutic strategies to overcome them.

Mutated gene/aberration	Disease	Mechanism	Possible therapeutic strategy	Ref.
*BTK*	CLL, MCL, WM, MZL	reversible binding of ibrutinib	third-generation BTK inhibitors, PROTAC-BTK, inhibitors of LYN and SYK	(7, 68–77)
*PLCG2*	CLL, MCL, WM, MZL	BTK-independent activation	inhibitors of RAC2, LYN, and SYK	(7, 68–71, 78, 79)
*CARD11*	CLL, MCL, WM, DLBCL, FL	↑ NFκB	proteasome or MALT1 inhibitor	(12, 71, 80–83)
*BIRC3, TRAF2, TRAF3*	MCL	↑ NFκB	MP3K14 inhibitor	(84, 85)
*CCND1*	MCL	cell cycle progression	unknown	(86)
*CDKN2A* and *MTAP* co-deletion	MCL	cell cycle progression	PRMT5 inhibitor	(87)
*SMARCA2*, *SMARCA4*, *ARID2*	MCL	disruption of SWI-SNF complex; ↑ BCL_XL_	BCL_XL_ inhibitor	(88)
*MYD88*^mt^/*CD79B*^wt^	DLBCL	MYD88-dependent and BCR-independent subtype	SYK or STAT3 inhibitor	(9, 89, 90)
*KLHL14*	DLBCL	↑ MYD88-TLR9-BCR super-complex	inhibition of BCR-dependent NFκB activation/mTOR inhibitors	(91)
*TNFAIP3*	DLBCL	↑ NFκB	unknown	(82)
2p+	CLL	↑XPO1	XPO1 inhibition (selinexor)	(92)
Del 8p	CLL	Loss of *TRAIL-R*, insensitivity to TRAIL-induced apoptosis	unknown; possibly venetoclax	(93)
Del 6q	WM	↑ MYD88/NFκB, loss of regulators of apoptosis	unknown	(94, 95)
Del 8p	WM	↑ TLR/MYD88, loss of *DOK2*, *BLK* and *TNFRSF10A/B*	unknown	(94)

↑ represents pathway/gene activation or upregulation.

“Del” stands for deletion of a chromosomal region.

Mutations in *BTK* and *PLCG2* have also been found in MCL and WM patients with acquired ibrutinib resistance as well as in one MZL patient (69–71). List of the most common *BTK* and *PLCG2* mutations is provided in the **Supplementary Table 2**. Though CLL cells on ibrutinib have a decreased NFκB binding to DNA elements, activating an alternative NFκB pathway by genetic changes is another mechanism responsible for ibrutinib resistance, mostly in MCL (65). This is caused by mutations in *BIRC3*, *TRAF2*, or *TRAF3*, whose absence leads to MP3K14 enzyme stabilization and constitutive activation of alternative NFκB pathway (**Figure 1**) (84, 85, 108, 109). Recurrent mutations in MCL patients who have relapsed on ibrutinib have also been found in *CARD11*, a protein responsible for BCR-induced NFκB activation, or in *CCND1*, a cyclin that promotes G1-S cell cycle progression (24, 81, 86). *CDKN2A* and *MTAP* co-deletion was also observed in ibrutinib-resistant MCL tumors (87). Additionally, loss and/or mutations in the SWI-SNF chromatin remodeling complex lead to the upregulation of anti-apoptotic BCL_XL_ and cause a primary or acquired resistance to the combination of ibrutinib and venetoclax in MCL (88).

In Waldenström’s macroglobulinemia (WM), mutations in *CARD11* also lead to ibrutinib resistance, and in WM patients the ibrutinib resistance may be accompanied by 6q and 8p chromosome region deletions that expand from pre-existing clones or emerge during treatment (71, 94). These chromosomal regions contain important signaling pathway regulators. The 6q region loss involves negative regulators of MYD88/NFκB (*TNFAIP3, HIVEP2, TRAF3IP2, IRAK1BP1*), an inhibitor of BTK (*IBTK*), and regulators of apoptosis (*FOXO3, BCLAF1, PERP*). The genes deleted on 8p include *DOK2*, a TLR/MYD88 signaling inhibitor, *BLK*, another target of ibrutinib that is important for B cell proliferation and differentiation, and *TNFRSF10A/B*, a gene encoding for TRAIL receptor (94, 95). Common mutations in WM are WHIM-like mutations in *CXCR4* and L265P mutations in *MYD88*, a mediator of Toll-like receptor signaling (110). In WM, *MYD88*^L265P^ activates NFκB *via* BTK and IRAK1/4, making these cells sensitive to ibrutinib (110, 111). WHIM-like mutations in *CXCR4* are responsible for impaired CXCR4 internalization upon stimulation and result in constant Akt and Erk activation. However, mutations in *MYD88*, which occur in 90% of WM patients, seem to have a more profound effect on the WM cell survival than *CXCR4*^WHIM^, as MYD88 inhibition could not be rescued by the *CXCR4*^WHIM^ mutation (112). These facts might explain why, even though WHIM-like mutations promote ibrutinib resistance *in vitro*, *MYD88*^wt^/*CXCR4*^wt^ patients have a lower response rate to ibrutinib therapy than *MYD88*^L265P^/*CXCR4*^wt^ or *MYD88*^L265P^/*CXCR4*^WHIM^ patients (13, 113).

In DLBCL, ibrutinib seems to be more effective in patients with an activated B cell-like DLBCL (ABC-DLBCL) subtype rather than in patients with germinal center B cell-like DLBCL (GC-DLBCL) due to constitutively active BCR signaling in ABC-DLBCL. However, even amongst ABC-DLBCL, complete or partial response was only detected in 37% of patients (9, 82), and a phase III clinical trial confirmed the benefit of adding ibrutinib to R-CHOP (rituximab plus cyclophosphamide, doxorubicin, vincristine, and prednisone) therapy only in younger patients with non-GC-DLBCL (114). As for resistance, it has been shown that DLBCL patients carrying mutations in *MYD88* and simultaneously having wild-type *CD79B* are primarily resistant to ibrutinib. As other combinations (*MYD88*^mt^/*CD79B*^mt^, *MYD88*^wt^/*CD79B*^mt^, *MYD88*^wt^/*MYD88*^wt^) are sensitive to ibrutinib, there is a possibility of an MYD88-dependent but BCR-independent ABC-DLBCL subtype (9). These findings might be explained by the formation of a multiprotein super-complex consisting of MYD88, TLR9 and BCR. It activates the NFκB pathway and is found in ABC-DLBCL tumors that respond to ibrutinib (**Figure 1**) (115). On the other hand, cells with inactivated *KLHL14*, a negative BCR component regulator often mutated in DLBCL, induce the NFκB pathway by activating the MYD88-TLR9-BCR super-complex, which partially protects them from ibrutinib-induced cell death (91). The role of knockout of the *KLHL14* tumor suppressor was demonstrated in rescuing the ABC-DLBCL cell line from apoptosis when IgM and CD79A were knocked down, which is a manipulation normally lethal to the DLBCL cells (91). Lastly, the above mentioned mutations in *CARD11* and inactivating mutations in *TNFAIP3*, a negative NFκB regulator, were also found in DLBCL patients not responding to ibrutinib treatment (82).

The genetic mechanisms of resistance, especially the *BTK* mutations that develop during ibrutinib therapy, are a clear indication of the on-target effects of the utilized small molecular inhibitors. The other mutations in the BCR signaling pathway members such as *PLCG2* or *CARD11* also demonstrate that malignant B cells try to gain or lose gene activity in order to overcome the inhibition of the key BCR-associated kinase (**Table 1**). However, it has been shown that cancer cells can also utilize non-genetic mechanisms to bypass the inhibition of a key pathway (116–120). In the following section we will review these potential mechanisms for ibrutinib/BTK-inhibition based therapy, and suggest the implications for combinatorial therapeutic strategies (**Table 2**).

**Table 2:** Non-genetic mechanisms of resistance/adaptation to ibrutinib and possible therapeutic strategies to overcome them.

Mechanism of resistance/adaptation	Disease	Possible therapeutic strategy	Ref.
↑ PI3K-Akt pathway	CLL, MCL, DLBCL	PI3K, mTOR, or XPO1 inhibitor	(92, 115, 121–132)
↑ JAK-STAT	CLL	Dual SYK/JAK-STAT inhibitor (Cerdulatinib)	(133)
↑ MYC	MCL	HSP90 inhibitor	(134)
↑ MAPK pathway	CLL, MCL, DLBCL	MEK inhibitor	(125, 135, 136)
↑ BCL2	CLL, DLBCL	BCL2 inhibitor (venetoclax)	(30, 125, 129, 137–143)
Metabolic reprogramming	CLL, MCL	OXPHOS inhibitor, inhibitor of fatty acid oxidation	(87, 144)
Integrin-mediated protection	CLL, MCL	VLA4 inhibition (FAK inhibitor)	(45, 145)
Resistant cancer stem cells	MCL	Wnt pathway inhibitor	(146)

↑ represents pathway/gene activation or upregulation.

## Non-Genetic Mechanisms of Adaptation to Ibrutinib

Patients resistant to ibrutinib that harbor the above-mentioned mutations often have these mutations subclonally in a relatively small fraction of surviving malignant B cells. Malignant non-mutated cells that co-exist with *BTK*^C418S^ mutated cells might be protected by the mutated cells though this has only been conceptually demonstrated in *MYD88*^mt^ WM and ABC-DLBCL. Mutated cells showed Erk1/2 activation, which led to pro-survival chemokine release and protection of *BTK*^wt^ cells (147). Even if this could explain the survival of the non-mutated sub-populations of cells, there are still ibrutinib-resistant patients (>20% of ibrutinib-resistant patients in CLL) that do not show any genetic mutations responsible for the resistance (96). This, together with slow cell apoptosis during ibrutinib therapy, suggest that there are non-genetic mechanisms of resistance and cells are able to partially adapt to ibrutinib and BTK inhibition (**Table 2**, **Figure 1**). Collectively, it seems that malignant B cells activate BTK-independent compensatory survival pathways under ibrutinib treatment, mainly PI3K/mTOR/Akt pathway and adhesion. Activating the NFκB pathway also induces some degree of ibrutinib resistance. This was described mainly by genetic mechanisms as mentioned before, but the non-canonical NFκB pathway together with MAPK signaling can also be activated and thus protect the cells from ibrutinib in MCL cells by activating CD40 (148, 149).

Activating the PI3K pathway is a well-known mechanism that rescues BCR deficient mature B cells from apoptosis (150, 151). It is therefore not surprising that more and more studies see a similar mechanism in malignant B cells by which they overcome BTK inhibition. Activated Akt has been observed in ibrutinib-resistant CLL and DLBCL cell lines, together with downregulated FoxO3a and PTEN levels (**Figure 1**) (121). Activating Akt/MAPK *via* CD79B overexpression has been sufficient to induce ibrutinib resistance in primary ABC-DLBCL (152). Ibrutinib also synergizes with CRISPR-Cas9 knockout of PI3Kδ (91). PI3K pathway activation was observed in B cell lymphoma patient-derived xenograft models with acquired resistance to ibrutinib and the growth of these tumors was blocked by combination of ibrutinib and idelalisib (122).

However, activation of PI3K/mTOR/Akt signaling after ibrutinib has been best described in MCL. Activated Akt and Erk levels, but not BTK, correlate with the response to ibrutinib in MCL cell lines and furthermore, ibrutinib-responsive patients have dephosphorylated Akt as opposed to non-responsive patients (69, 153). Ibrutinib in MCL strengthens cell adhesion *via* integrin dimer VLA4 formed by β1-integrin and CD49d and activates the PI3K/Akt pathway in this way (**Figure 1**). Zhao *et al*. described this phenomenon by showing that ibrutinib-resistant cells have a higher β1-integrin expression that helps to form an ILK-Rictor complex that activates a pro-survival mTORC2/Akt pathway. This was disrupted by ibrutinib in combination with dual mTOR1/2 inhibitor AZD8055 or dual PI3K-mTOR1/2 inhibitor BEZ235 (123). Here, it is also worth mentioning that ibrutinib-resistant MCL samples upregulate the mTOR signaling pathway (as well as genes involved in cell cycle regulation and MYC targets) compared to ibrutinib-sensitive cells (87). A similar compensatory survival mechanism was seen by Guan and colleagues who demonstrated that stromal cells protect MCL cells from ibrutinib-induced death *via* their interaction with VLA4. A combination of ibrutinib with VLA4 blockage or with an inhibitor of PI3K catalytic p110α subunit disrupted the interaction and overcame the resistance (124).

In CLL, the cells lose the ability to adhere to fibronectin almost completely and partly to stromal cells when treated with ibrutinib *in vitro* for a short period of time (63, 154). Interestingly though, BCR stimulation activates VLA4 in CLL cells exposed to ibrutinib for an extended time *via* a BTK-independent manner involving PI3K. Also, higher CD49d levels in patients prevent ibrutinib-induced lymphocytosis and cause a lower nodal response. This translates into a shorter progression-free survival in ibrutinib treated patients (42). Analogically, it has been described that ibrutinib induces CXCR4 expression on cell-surface, which might translate to a paradoxically increased responsiveness of these cells to chemokine ligands, however, it is likely that the response to chemokines in this context is not completely physiological since BTK is involved in the chemokine pathway (30, 155). Time to progression can also be predicted by cell-surface IgM levels prior to ibrutinib treatment, suggesting another non-genetic mechanism of ibrutinib resistance in CLL, potentially similar to the CD79B overexpression in DLBCL (**Figure 1**) (152, 156). In CLL, the mechanism might be explained by BCR signaling bypassing BTK upon BCR crosslinking as ibrutinib is not able to properly inhibit Ca^2+^ mobilization and Erk1/2 phosphorylation when surface IgM levels are high (156). Moreover, cell-surface IgM levels rise during ibrutinib treatment in CLL patients, although this might depend on the time since the start of therapy (30, 157).

Activating the MAPK pathway might also be one of the compensatory mechanisms for BTK inhibition, as noted in a recent study. Upregulation of the genes involved in MAPK was observed by Forestieri et al. in residual CLL cells after ibrutinib treatment and in addition, acquired mutations in *BRAF*, *NRAS*, and *KRAS* were found in a fraction of patients (135). This might explain the observed synergy between MEK inhibitors with “BCR inhibitors” in B cell lymphomas (125, 136).

Besides the described pathways, other ibrutinib resistance mechanisms are also possible and fit the biology of B cells. It has been shown that MYC acts as a key downstream BCR effector, and its over-expression can rescue the absence of BCR activity in some B cells (158, 159). Indeed, upregulation of MYC has been observed in ibrutinib-resistant MCL cell lines and this resistance can be reversed by inhibiting HSP90 (134). Protection from ibrutinib can also be provided by cells in the microenvironment (133, 160, 161). On the other hand, CLL cells resistant to BTK inhibition recover the ability to produce and respond to IL4 and require less T cell help for growth (162). Lymphoma relapse can potentially also arise from cancer stem cells described in MCL and FL. Their quiescent phenotype, together with high ABC-transporter activity gives them general drug-resistant properties (163–166). Indeed, the MCL-initiating cells were found to be resistant to ibrutinib and could be eliminated by inhibiting Wnt signaling pathway whose genes were overexpressed in these cancer stem cells (146).

## Targeting Ibrutinib Resistance

There are several potential ways to overcome ibrutinib resistance such as i) in cases with specific *BTK* mutations using third generation BTK inhibitors which do not target C481, or PROTAC mediated BTK degradation, ii) using different molecular targets once a patient is resistant to ibrutinib, or iii) preventing the resistance by a more rapid B cell elimination that would lower the chance of developing resistance or activating compensatory survival pathways (**Figure 1**, **Tables 1** and **2**). Additionally, time-limited or more selective treatment would likely lower toxicities in patients as up to 40% of CLL patients discontinue ibrutinib therapy, which is caused mostly by the toxicities (11, 167).

Unfortunately, second-generation BTK inhibitors such as acalabrutinib, zanubrutinib, or tirabrutinib are not able to overcome the resistance caused by *BTK*^C481S^ since they bind to the same protein region as ibrutinib. Their advantage is their higher selectivity with less off-targets (**Supplementary Table 1**) and lesser toxicities than ibrutinib, making them more feasible for patients intolerant to ibrutinib (168–172). Acalabrutinib has recently been approved for CLL and MCL and zanubrutinib for MCL (171, 173). The solution to *BTK*^C481S^ mutations could be the use of non-covalent third-generation BTK inhibitors that are able to inhibit the kinase’s activity independently of C481S (**Supplementary Table 1**). BTK inhibitors such as fenebrutinib (GDC-0853), LOXO-305, or vecabrutinib are currently in the early phases of clinical testing even for patients with *BTK*^C481S^ (72–74). ARQ 531 has shown better efficacy than ibrutinib in murine models resembling Richter transformation, targets CLL not only with *BTK* but also *PLCG2* mutations and has off-target activity against kinases in Erk signaling and kinases in the Src family (75). The off-targets of various BTK inhibitors are summarized in the **Supplementary Table 1**. A different but also promising therapeutic strategy is provided by PROTAC which degrades its target with E3 ligase. PROTAC-induced BTK degradation is highly selective and effective in treating *BTK*^C481S^ ibrutinib-resistant mouse models (76, 77). CLL cells with R665W and L845F mutations in *PLCG2* are sensitive to RAC2 or SYK and LYN inhibition (78, 79). Inhibiting RAC2 or its binding partner VAV1 is synergistic with ibrutinib also in DLBCL just like inhibiting STAT3 or SYK together with ibrutinib in *MYD88* mutated DLBCL (89–91).

Another approach would be combining ibrutinib with compensatory survival pathway inhibitors such as PI3K/mTOR/Akt or NFκB (**Table 2**). As mentioned before, ibrutinib synergizes with PI3K/mTOR/Akt pathway inhibitors in MCL, CLL and DLBCL (121–126). A combination of ibrutinib and umbralisib, a next-generation PI3Kδ inhibitor, was studied in a clinical trial in CLL and MCL patients with promising results (127). It has been shown that ibrutinib increases CLL-cell sensitivity to mTOR inhibitors as well as proteasome and PLK1 inhibitors (128). Targeting mTOR combined with ibrutinib was also suggested in ABC-DLBCL by Phelan et al. as mTORC1/2 inhibitor AZD2014 further attenuates formation of MYD88/TLR9/BCR super-complex when compared to ibrutinib alone (115). CC-115, a dual mTOR/DNA-dependent protein kinase inhibitor, is now in a clinical trial and is able to revert CD40-mediated resistance to venetoclax and also inhibits BCR signaling in CLL patients with acquired idelalisib resistance (174). Inhibiting the PI3K/mTOR/Akt pathway has been shown to be successful also in *in vitro* drug screening in DLBCL, where PI3K inhibitors synergized with ibrutinib (129). The same study, confirmed by others, proved a synergy between ibrutinib and inhibition of IRAK4, a mediator for TLR and NFκB activation whose targeting is studied not only in DLBCL but in CLL as well (129, 175, 176). Furthermore, the synergy with ibrutinib was also seen in combination with selinexor, an XPO1 inhibitor, although this seems to be regulated again *via* the PI3K/mTOR/Akt pathway as selinexor restores a nuclear abundance of FoxO3a and PTEN after ibrutinib treatment, resulting in the inhibition of PI3K/mTOR/Akt signaling activation (**Figure 1**) (121, 129). Absence of tumor suppressors FoxO3a and PTEN in the nucleus could be an explanation for ibrutinib resistance in 2p+ CLL patients who overexpress *XPO1* and why selinexor and next-generation XPO1 inhibitors seem to be efficient in preclinical CLL and MCL models where it reduces NFκB binding to DNA (92, 130–132). Another molecule that could be targeted together with BTK is the MP3K14 enzyme, a member of an alternative NFκB pathway constitutively activated in ibrutinib-resistant MCL patients due to mutations in *BIRC3*, *TRAF2*, or *TRAF3* (**Figure 1**) (84, 85).

An attractive strategy to increase the treatment efficacy is combining drugs that are already approved in therapy. Recently, ibrutinib has been shown to be efficient with venetoclax, a BH3 mimetic that inhibits anti-apoptotic molecule BCL2 and is approved for CLL treatment (125, 137, 138, 177). This combination is rational as ibrutinib induces BCL2 expression and on the other hand, decreases anti-apoptotic MCL1 levels, which can be a cause of venetoclax resistance (**Figure 1**) (30, 139–141). Higher BCL2 levels have also been found in DLBCL patients with poorer response to ibrutinib therapy (142). Furthermore, ibrutinib inhibits malignant cell proliferation while venetoclax targets preferentially resting sub-populations, potentially explaining the synergy of these two drugs in B cell malignancies (129, 143). Combining ibrutinib with fludarabine, a purine analog commonly used together with cyclophosphamide and anti-CD20 monoclonal antibody rituximab to treat CLL patients, has been shown to be synergic *ex vivo* (125). Ibrutinib with immune modulator lenalidomide and rituximab is under investigation in DLBCL and MCL but has not been successful in CLL (178–181). A profoundly studied possibility for therapy is combining “BCR inhibitors” with widely-used anti-CD20 monoclonal antibodies (28). However, adding rituximab to ibrutinib did not bring any clinical benefit and this is likely due to ibrutinib downregulating CD20 levels and/or interfering with effector cell functions (28, 30, 182, 183). Furthermore, ibrutinib has been shown to negatively regulate anti-CD20 induced apoptosis in MCL cell lines (184). A combination of ibrutinib with a more efficient anti-CD20 antibody, obinutuzumab, is now approved for CLL therapy. However, the real benefit of obinutuzumab still remains unclear as the control arm of the clinical trial was chlorambucil with obinutuzumab (185). In the ELEVATE clinical trial, acalabrutinib or acalabrutinib plus obinutuzumab were both superior to chlorambucil plus obinutuzumab (170). Even though it is not yet clear whether this combination reduces the occurrence of acquired BTK inhibition resistance, it is true that re-distributing malignant cells to the peripheral blood makes malignant cells more susceptible to monoclonal antibodies (145, 186). An interesting option is ibrutinib combined with anti-ROR1 monoclonal antibody in CLL, which is expected to have a great specificity for malignant B cells, and ROR1 levels are not reduced during ibrutinib therapy (187). Promising results have also been obtained from a fixed-duration treatment with venetoclax and obinutuzumab in CLL (188). Therapy nowadays aims for a time-limited treatment setting as it would lower the selection pressure, leave shorter time for cells to compensate for inhibition of targeted pathway, and reduce toxicities in patients.

It has been reported that patients who relapse or are intolerant to one kinase-inhibitor benefit from a change to different small-molecule inhibitors rather than chemotherapy (189). Novel therapeutic targets and drugs are therefore being investigated. Promising results have been obtained by targeting different players in BCR signaling such as PKCβ or MALT1 whose inhibition is effective in ABC-DLBCL, MCL, and naïve as well as ibrutinib-resistant CLL (190–193). Cerdulatinib, a dual SYK/JAK-STAT inhibitor, targets BCR signaling and is also able to overcome microenvironmental protection and blocks proliferation in ibrutinib-resistant primary CLL samples and BTK^C481S^ lymphoma cell lines (**Figure 1**) (133). Directly targeting microenvironmental interactions, migration and adhesion could also have potential use in therapy of ibrutinib-resistant patients *via* the use of natalizumab or CXCR4 inhibitor plerixafor (45, 145). Lastly, malignant cells of ibrutinib-resistant CLL and MCL patients show metabolic reprogramming, which has also been suggested as a possible therapeutic target (87, 144).

## Effects of Idelalisib and Mechanisms of Resistance

Another molecule in BCR signaling widely therapeutically targeted in B cell malignancies is PI3K. Activated PI3K/Akt axis in B cell malignancies and its signaling pathway’s role in cell survival makes it an attractive therapeutic target (3, 151, 194, 195). PI3K exists in four catalytic isoforms: p110α, p110β, p110γ, and p110δ. p110α (PI3Kα) and p110δ (PI3Kδ) are both needed for “tonic” (antigen-independent) BCR signaling, while only p110δ is needed for antigen-induced BCR signaling (196). The PI3Kδ isoform is targeted by widely-used idelalisib, which has been approved for the treatment of CLL, FL, and non-Hodgkin lymphomas. Idelalisib not only thwarts the PI3K/Akt/mTOR pathway but also inhibits cell migration towards chemokines and adhesion to stromal cells, which, just like with ibrutinib, leads to an initial increase in the number of lymphocytes in the peripheral blood caused by lymphocyte migration out of the tissues in CLL. This is accompanied by a reduction in Akt phosphorylation and other downstream effectors as well as by apoptosis induction (8, 197–199). Unfortunately, due to serious adverse effects and infections, it has been suggested to primarily give idelalisib to CLL patients with progression on ibrutinib or indolent NHL patients with progression on two prior therapies (14, 200, 201). As already mentioned, promising results have recently been seen in a clinical trial of a combination of ibrutinib and a next-generation dual PI3Kδ/CK1ϵ inhibitor, umbralisib (127). Furthermore, the importance of the p110γ subunit is now emerging in CLL. Its activation does not respond to BCR stimulation but increases in response to CD40L/IL4 and cells with overexpressed PI3Kγ show an enhanced cell migration towards CXCL12. A dual PI3Kδ/γ inhibitor, duvelisib, seems to have a bigger impact on cell migration than idelalisib alone and is now approved to treat CLL/SLL and FL (197). Another PI3K inhibitor, copanlisib, is approved for relapsed/refractory FL. It is a pan-class I PI3K inhibitor that inhibits all four PI3K isoforms with higher selectivity against PI3Kα and PI3Kδ (202). Its advantage lies not only in its molecular mechanism as inhibition of other PI3K isoforms can lower viability and migration of B cells, but it also seems to cause fewer adverse events in patients when compared to idelalisib (197, 203–205).

Despite idelalisib’s and other PI3K inhibitors’ ability to initially control the disease in some patients, a fraction of patients develop resistance. Unfortunately, unlike in ibrutinib treated patients, the mechanisms of resistance remain mostly unclear (8, 206). No recurrent mutations were found in patients progressing on idelalisib nor have they been found in a mouse model resistant to PI3Kδ inhibition (207, 208). Two studies have shown the role of MAPK signaling in resisting PI3Kδ inhibition. Firstly, Murali et al. confirmed activating mutations in MAPK pathway leading to Erk phosphorylation in patients resistant to PI3K inhibition and suggested that blocking Erk might sensitize patients to PI3K inhibitors (**Figure 1**) (209). Secondly, a non-genetic mechanism of resistance was found in PI3Kδ resistant mice, where the upregulation of insulin-like growth factor 1 receptor (IGF1R) led to MAPK signaling activation. IGF1R upregulation was caused by FoxO1 and GSK3β and the resistance was resolved by inhibiting the receptor with linsitinib (208). Initial experiments with copanlisib point to IL6 signaling as a main player in copanlisib resistance; levels of IL6 and phosphorylation of STAT5, Akt, p70S6K, and MAPK were increased in copanlisib-resistant B cell lymphoma cell lines and the resistance was reversible by JAK inhibitor (210).

Extensive research is needed in order to reveal the resistance mechanisms for PI3K inhibitors and it seems that they might be more complex than in BTK inhibition. Despite their adverse effects in patients and the emergence of BCL2 inhibitors and next-generation BTK inhibitors, the PI3K/mTOR/Akt signaling pathway plays an important role in B cell malignancy pathogenesis and its inhibition might find its therapeutic place.

## Discussion

Targeting BCR signaling is now a commonly used therapy strategy for B cell malignancies. Unfortunately, some patients are primarily resistant to “BCR inhibitors” or develop resistance during the course of treatment. Furthermore, the now required continuous treatment often leads to toxicities and forces a change of therapy. *BTK* and *PLCG2* mutations are the most common and best-described mechanisms of resistance to BTK inhibitor ibrutinib, although recurrent mutations in other genes or aberrations in larger chromosomal regions have been described as being responsible for the resistance across the B cell malignancies. Interestingly, malignant B cells are able to overcome BTK inhibition by non-genetic mechanisms as well. These include activation of compensatory survival pathway, such as PI3K/mTOR/Akt, NFkB, or MAPK signaling pathways. Compensatory survival is also provided by the upregulation of anti-apoptotic BCL2, MYC or adhesion involved integrins. Even though a lot is known about BTK inhibition, PI3K inhibitor resistance remains largely unclear. Several studies point to MAPK pathway activation as a compensatory mechanism to PI3K inhibition, but further research is needed in this area.

Resistance caused by *BTK* mutations can be solved by third-generation BTK inhibitors now in clinical trials or by BTK degradation by PROTAC-based compounds; mutations in *PLCG2* by combining BTK inhibition with RAC2 or SYK and LYN inhibition. Compensatory survival by upregulating other signaling pathways could be solved by combining several inhibitors as well. The synergy between blockage of BTK and the PI3K/mTOR/Akt pathway has been shown repeatedly in CLL, DLBCL and MCL. It remains largely unknown how the non-coding part of the genome influencing BCR signaling is affected by “BCR inhibitors” and if (de)regulation of these molecules could contribute to therapy resistance or be directly used as a therapeutic target (20, 31, 34, 35). Novel therapeutic targets and strategies are still being investigated and their inhibition is tested alone or in combination with “BCR inhibitors”. These combinations should be supported by analyzing the responses of malignant cells to individual drugs and using multi-omics to identify possible compensatory signaling pathways to be co-targeted by small-molecule inhibitors. Furthermore, co-targeting two kinases in seemingly the same pathway can also have a synergistic effect, as observed by BTK and PI3K inhibition’s synergy. Analyzing individual patient-to-patient response to “BCR inhibitors” could help to identify specific compensatory pathways and this could help to define a personalized combinatorial therapy. However, it is likely that there might be some universal responses to “BCR inhibitors” in each B cell malignancy and this could follow pre-existing mechanisms that allow B cells to survive for an extended time without antigen encounter. Single cell analysis of the response to BTK/PI3K inhibitors in multiple patients could help to understand if the response or adaptation to therapy follows a generally uniform course or if there are major intra- and inter- patient specific mechanisms. Besides transcriptomics and proteomics, research should focus on describing the cells’ immunophenotypic profiles after “BCR inhibition” to identify surface molecules that could be targeted by monoclonal antibodies, since this can be of high specificity for cancer cells and low toxicity in general. The time dynamics of changes during therapy should also be studied in order to describe the mechanisms of early and long-term adaptation and potentially identify an optimal time point for adding a second drug in combination. Improving clinical efficacy of drug combinations containing “BCR inhibitors” should allow a time-limited treatment with a deep molecular response, decreased chance of resistance and limited toxicities associated with long-term therapy.

## Author Contributions

LO and MM wrote the manuscript. All authors contributed to the article and approved the submitted version.

## Funding

This work was supported by the Czech Science Foundation (project No. 20-02566S). This project has received funding from the European Research Council (ERC) under the European Union’s Horizon 2020 research and innovation programme (grant agreement No 802644). Supported by MH CZ - DRO (FNBr, 65269705). This research was carried out under the project CEITEC 2020 (LQ1601) with financial support from the Ministry of Education, Youth and Sports of the Czech Republic under the National Sustainability Programme II. LO is a Brno Ph.D. Talent Scholarship Holder - Funded by the Brno City Municipality.

## Conflict of Interest

The authors declare that the research was conducted in the absence of any commercial or financial relationships that could be construed as a potential conflict of interest.

## References

[1] Devan J Janikova A Mraz M New concepts in follicular lymphoma biology: From BCL2 to epigenetic regulators and non-coding RNAs. Semin Oncol (2018) 45:291–302. 10.1053/j.seminoncol.2018.07.005 30360879

[2] Dühren-von Minden M Übelhart R Schneider D Wossning T Bach MP Buchner M Chronic lymphocytic leukaemia is driven by antigen-independent cell-autonomous signalling. Nature (2012) 489:309–12. 10.1038/nature11309 22885698

[3] Ringshausen I Schneller F Bogner C Hipp S Duyster J Peschel C Constitutively activated phosphatidylinositol-3 kinase (PI-3K) is involved in the defect of apoptosis in B-CLL: association with protein kinase Cdelta. Blood (2002) 100:3741–8. 10.1182/blood-2002-02-0539 12393602

[4] Saba NS Liu D Herman SEM Underbayev C Tian X Behrend D Pathogenic role of B-cell receptor signaling and canonical NF-κB activation in mantle cell lymphoma. Blood (2016) 128:82–92. 10.1182/blood-2015-11-681460 27127301 PMC4937360

[5] Seda V Mraz M. B-cell receptor signalling and its crosstalk with other pathways in normal and malignant cells. Eur J Haematol (2015) 94:193–205. 10.1111/ejh.12427 25080849

[6] Lew TE Anderson MA Seymour JF Promises and pitfalls of targeted agents in chronic lymphocytic leukemia. Cancer Drug Resist (2020) 3:415–44. 10.20517/cdr.2019.108 PMC8992498 35582452

[7] Woyach JA Furman RR Liu T-M Ozer HG Zapatka M Ruppert AS Resistance mechanisms for the Bruton’s tyrosine kinase inhibitor ibrutinib. N Engl J Med (2014) 370:2286–94. 10.1056/NEJMoa1400029 PMC4144824 24869598

[8] Brown JR Byrd JC Coutre SE Benson DM Flinn IW Wagner-Johnston ND Idelalisib, an inhibitor of phosphatidylinositol 3-kinase p110δ, for relapsed/refractory chronic lymphocytic leukemia. Blood (2014) 123:3390–7. 10.1182/blood-2013-11-535047 PMC4123414 24615777

[9] Wilson WH Gerecitano JF Goy A de Vos S Kenkre VP Barr PM The Bruton’s Tyrosine Kinase (BTK) Inhibitor, Ibrutinib (PCI-32765), Has Preferential Activity in the ABC Subtype of Relapsed/Refractory De Novo Diffuse Large B-Cell Lymphoma (DLBCL): Interim Results of a Multicenter, Open-Label, Phase 2 Study. Blood (2012) 120:686–6. 10.1182/blood.V120.21.686.686

[10] Jain P Kanagal-Shamanna R Zhang S Ahmed M Ghorab A Zhang L Long-term outcomes and mutation profiling of patients with mantle cell lymphoma (MCL) who discontinued ibrutinib. Br J Haematol (2018) 183:578–87. 10.1111/bjh.15567 30175400

[11] Mato AR Nabhan C Thompson MC Lamanna N Brander DM Hill B Toxicities and outcomes of 616 ibrutinib-treated patients in the United States: a real-world analysis. Haematologica (2018) 103:874–9. 10.3324/haematol.2017.182907 PMC5927982 29419429

[12] Bartlett NL Costello BA LaPlant BR Ansell SM Kuruvilla JG Reeder CB Single-agent ibrutinib in relapsed or refractory follicular lymphoma: a phase 2 consortium trial. Blood (2018) 131:182–90. 10.1182/blood-2017-09-804641 PMC5757691 29074501

[13] Treon SP Tripsas CK Meid K Warren D Varma G Green R Ibrutinib in Previously Treated Waldenström’s Macroglobulinemia. New Engl J Med (2015) 372:1430–40. 10.1056/NEJMoa1501548 25853747

[14] Salles G Schuster SJ de Vos S Wagner-Johnston ND Viardot A Blum KA Efficacy and safety of idelalisib in patients with relapsed, rituximab- and alkylating agent-refractory follicular lymphoma: a subgroup analysis of a phase 2 study. Haematologica (2017) 102:e156–9. 10.3324/haematol.2016.151738 PMC5395130 27979923

[15] Rolli V Gallwitz M Wossning T Flemming A Schamel WWA Zürn C Amplification of B cell antigen receptor signaling by a Syk/ITAM positive feedback loop. Mol Cell (2002) 10:1057–69. 10.1016/S1097-2765(02)00739-6 12453414

[16] Aiba Y Kameyama M Yamazaki T Tedder TF Kurosaki T Regulation of B-cell development by BCAP and CD19 through their binding to phosphoinositide 3-kinase. Blood (2008) 111:1497–503. 10.1182/blood-2007-08-109769 18025150

[17] Okada T Maeda A Iwamatsu A Gotoh K Kurosaki T BCAP: the tyrosine kinase substrate that connects B cell receptor to phosphoinositide 3-kinase activation. Immunity (2000) 13:817–27. 10.1016/S1074-7613(00)00079-0 11163197

[18] Sarbassov DD Guertin DA Ali SM Sabatini DM Phosphorylation and regulation of Akt/PKB by the rictor-mTOR complex. Science (2005) 307:1098–101. 10.1126/science.1106148 15718470

[19] Ingham RJ Santos L Dang-Lawson M Holgado-Madruga M Dudek P Maroun CR The Gab1 docking protein links the b cell antigen receptor to the phosphatidylinositol 3-kinase/Akt signaling pathway and to the SHP2 tyrosine phosphatase. J Biol Chem (2001) 276:12257–65. 10.1074/jbc.M010590200 11278704

[20] Mraz M Chen L Rassenti LZ Ghia EM Li H Jepsen K miR-150 influences B-cell receptor signaling in chronic lymphocytic leukemia by regulating expression of GAB1 and FOXP1. Blood (2014) 124:84–95. 10.1182/blood-2013-09-527234 24787006 PMC4125356

[21] Saito K Scharenberg AM Kinet JP Interaction between the Btk PH domain and phosphatidylinositol-3,4,5-trisphosphate directly regulates Btk. J Biol Chem (2001) 276:16201–6. 10.1074/jbc.M100873200 11279148

[22] Park H Wahl MI Afar DE Turck CW Rawlings DJ Tam C Regulation of Btk function by a major autophosphorylation site within the SH3 domain. Immunity (1996) 4:515–25. 10.1016/S1074-7613(00)80417-3 8630736

[23] Rawlings DJ Scharenberg AM Park H Wahl MI Lin S Kato RM Activation of BTK by a phosphorylation mechanism initiated by SRC family kinases. Science (1996) 271:822–5. 10.1126/science.271.5250.822 8629002

[24] Shinohara H Yasuda T Aiba Y Sanjo H Hamadate M Watarai H PKC beta regulates BCR-mediated IKK activation by facilitating the interaction between TAK1 and CARMA1. J Exp Med (2005) 202:1423–31. 10.1084/jem.20051591 PMC2212994 16301747

[25] Shinohara H Kurosaki T Comprehending the complex connection between PKCbeta, TAK1, and IKK in BCR signaling. Immunol Rev (2009) 232:300–18. 10.1111/j.1600-065X.2009.00836.x 19909372

[26] Kang SW Wahl MI Chu J Kitaura J Kawakami Y Kato RM PKCbeta modulates antigen receptor signaling via regulation of Btk membrane localization. EMBO J (2001) 20:5692–702. 10.1093/emboj/20.20.5692 PMC125669 11598012

[27] Chan VW Meng F Soriano P DeFranco AL Lowell CA Characterization of the B lymphocyte populations in Lyn-deficient mice and the role of Lyn in signal initiation and down-regulation. Immunity (1997) 7:69–81. 10.1016/S1074-7613(00)80511-7 9252121

[28] Pavlasova G Mraz M The regulation and function of CD20: an “enigma” of B-cell biology and targeted therapy. Haematologica (2020) 105:1494–506. 10.3324/haematol.2019.243543 PMC7271567 32482755

[29] Pavlasova G Borsky M Svobodova V Oppelt J Cerna K Novotna J Rituximab primarily targets an intra-clonal BCR signaling proficient CLL subpopulation characterized by high CD20 levels. Leukemia (2018) 32:2028–31. 10.1038/s41375-018-0211-0 30030508

[30] Pavlasova G Borsky M Seda V Cerna K Osickova J Doubek M Ibrutinib inhibits CD20 upregulation on CLL B cells mediated by the CXCR4/SDF-1 axis. Blood (2016) 128:1609–13. 10.1182/blood-2016-04-709519 PMC5291297 27480113

[31] Cerna K Oppelt J Chochola V Musilova K Seda V Pavlasova G MicroRNA miR-34a downregulates FOXP1 during DNA damage response to limit BCR signalling in chronic lymphocytic leukaemia B cells. Leukemia (2019) 33:403–14. 10.1038/s41375-018-0230-x 30111844

[32] Cerna K Mraz M p53 limits B cell receptor (BCR) signalling: a new role for miR-34a and FOXP1. Oncotarget (2018) 9:36409–10. 10.18632/oncotarget.26376 PMC6284860 30559925

[33] Cui B Chen L Zhang S Mraz M Fecteau J-F Yu J MicroRNA-155 influences B-cell receptor signaling and associates with aggressive disease in chronic lymphocytic leukemia. Blood (2014) 124:546–54. 10.1182/blood-2014-03-559690 PMC4110661 24914134

[34] Musilova K Devan J Cerna K Seda V Pavlasova G Sharma S miR-150 downregulation contributes to the high-grade transformation of follicular lymphoma by upregulating FOXP1 levels. Blood (2018) 132:2389–400. 10.1182/blood-2018-06-855502 30213873

[35] Musilova K Mraz M MicroRNAs in B-cell lymphomas: how a complex biology gets more complex. Leukemia (2015) 29:1004–17. 10.1038/leu.2014.351 25541152

[36] Olive V Bennett MJ Walker JC Ma C Jiang I Cordon-Cardo C miR-19 is a key oncogenic component of mir-17-92. Genes Dev (2009) 23:2839–49. 10.1101/gad.1861409 PMC2800084 20008935

[37] Palacios F Abreu C Prieto D Morande P Ruiz S Fernández-Calero T Activation of the PI3K/AKT pathway by microRNA-22 results in CLL B-cell proliferation. Leukemia (2015) 29:115–25. 10.1038/leu.2014.158 24825182

[38] Psathas JN Doonan PJ Raman P Freedman BD Minn AJ Thomas-Tikhonenko A The Myc-miR-17-92 axis amplifies B-cell receptor signaling via inhibition of ITIM proteins: a novel lymphomagenic feed-forward loop. Blood (2013) 122:4220–9. 10.1182/blood-2012-12-473090 PMC3868926 24169826

[39] Carrasco YR Batista FD. B-cell activation by membrane-bound antigens is facilitated by the interaction of VLA-4 with VCAM-1. EMBO J (2006) 25:889–99. 10.1038/sj.emboj.7600944 PMC1383545 16456548

[40] Buchner M Baer C Prinz G Dierks C Burger M Zenz T Spleen tyrosine kinase inhibition prevents chemokine- and integrin-mediated stromal protective effects in chronic lymphocytic leukemia. Blood (2010) 115:4497–506. 10.1182/blood-2009-07-233692 20335218

[41] Spaargaren M Beuling EA Rurup ML Meijer HP Klok MD Middendorp S The B cell antigen receptor controls integrin activity through Btk and PLCgamma2. J Exp Med (2003) 198:1539–50. 10.1084/jem.20011866 PMC2194118 14610042

[42] Tissino E Benedetti D Herman SEM Ten Hacken E Ahn IE Chaffee KG Functional and clinical relevance of VLA-4 (CD49d/CD29) in ibrutinib-treated chronic lymphocytic leukemia. J Exp Med (2018) 215:681–97. 10.1084/jem.20171288 PMC5789417 29301866

[43] Burger JA Tsukada N Burger M Zvaifler NJ Dell’Aquila M Kipps TJ Blood-derived nurse-like cells protect chronic lymphocytic leukemia B cells from spontaneous apoptosis through stromal cell-derived factor-1. Blood (2000) 96:2655–63. 10.1182/blood.V96.8.2655.h8002655_2655_2663 11023495

[44] Burger JA Burger M Kipps TJ Chronic lymphocytic leukemia B cells express functional CXCR4 chemokine receptors that mediate spontaneous migration beneath bone marrow stromal cells. Blood (1999) 94:3658–67. 10.1182/blood.V94.11.3658.423k11_3658_3667 10572077

[45] Burger M Hartmann T Krome M Rawluk J Tamamura H Fujii N Small peptide inhibitors of the CXCR4 chemokine receptor (CD184) antagonize the activation, migration, and antiapoptotic responses of CXCL12 in chronic lymphocytic leukemia B cells. Blood (2005) 106:1824–30. 10.1182/blood-2004-12-4918 15905192

[46] Aguilar-Hernandez MM Blunt MD Dobson R Yeomans A Thirdborough S Larrayoz M IL-4 enhances expression and function of surface IgM in CLL cells. Blood (2016) 127:3015–25. 10.1182/blood-2015-11-682906 27002119

[47] Steele AJ Prentice AG Cwynarski K Hoffbrand AV Hart SM Lowdell MW The JAK3-selective inhibitor PF-956980 reverses the resistance to cytotoxic agents induced by interleukin-4 treatment of chronic lymphocytic leukemia cells: potential for reversal of cytoprotection by the microenvironment. Blood (2010) 116:4569–77. 10.1182/blood-2009-09-245811 20716767

[48] Ghia P Strola G Granziero L Geuna M Guida G Sallusto F Chronic lymphocytic leukemia B cells are endowed with the capacity to attract CD4+, CD40L+ T cells by producing CCL22. Eur J Immunol (2002) 32:1403–13. 10.1002/1521-4141(200205)32:5&lt;1403::AID-IMMU1403<3.0.CO;2-Y 11981828

[49] Herishanu Y Pérez-Galán P Liu D Biancotto A Pittaluga S Vire B The Lymph Node Microenvironment Promotes B-cell Receptor Signaling, NF-kappaB Activation, and Tumor Proliferation in Chronic Lymphocytic Leukemia. Blood (2011) 117:563–74. 10.1182/blood-2010-05-284984 PMC3031480 20940416

[50] Ruan J Hyjek E Kermani P Christos PJ Hooper AT Coleman M Magnitude of stromal hemangiogenesis correlates with histologic subtype of non-Hodgkin’s lymphoma. Clin Cancer Res (2006) 12:5622–31. 10.1158/1078-0432.CCR-06-1204 17020964

[51] Burger JA Quiroga MP Hartmann E Bürkle A Wierda WG Keating MJ High-level expression of the T-cell chemokines CCL3 and CCL4 by chronic lymphocytic leukemia B cells in nurselike cell cocultures and after BCR stimulation. Blood (2009) 113:3050–8. 10.1182/blood-2008-07-170415 PMC4916945 19074730

[52] Bürkle A Niedermeier M Schmitt-Gräff A Wierda WG Keating MJ Burger JA Overexpression of the CXCR5 chemokine receptor, and its ligand, CXCL13 in B-cell chronic lymphocytic leukemia. Blood (2007) 110:3316–25. 10.1182/blood-2007-05-089409 17652619

[53] Slinger E Thijssen R Kater AP Eldering E Targeting antigen-independent proliferation in chronic lymphocytic leukemia through differential kinase inhibition. Leukemia (2017) 31:2601–7. 10.1038/leu.2017.129 28462919

[54] Chang BY Francesco M De Rooij MFM Magadala P Steggerda SM Huang MM Egress of CD19(+)CD5(+) cells into peripheral blood following treatment with the Bruton tyrosine kinase inhibitor ibrutinib in mantle cell lymphoma patients. Blood (2013) 122:2412–24. 10.1182/blood-2013-02-482125 PMC3790509 23940282

[55] Woyach JA Smucker K Smith LL Lozanski A Zhong Y Ruppert AS Prolonged lymphocytosis during ibrutinib therapy is associated with distinct molecular characteristics and does not indicate a suboptimal response to therapy. Blood (2014) 123:1810–7. 10.1182/blood-2013-09-527853 PMC3962160 24415539

[56] Satterthwaite AB Cheroutre H Khan WN Sideras P Witte ON Btk dosage determines sensitivity to B cell antigen receptor cross-linking. Proc Natl Acad Sci USA (1997) 94:13152–7. 10.1073/pnas.94.24.13152 PMC24278 9371815

[57] Tsukada S Saffran DC Rawlings DJ Parolini O Allen RC Klisak I Deficient expression of a B cell cytoplasmic tyrosine kinase in human X-linked agammaglobulinemia. Cell (1993) 72:279–90. 10.1016/0092-8674(93)90667-F 8425221

[58] Burger JA Li KW Keating MJ Sivina M Amer AM Garg N Leukemia cell proliferation and death in chronic lymphocytic leukemia patients on therapy with the BTK inhibitor ibrutinib. JCI Insight (2017) 2:e89904. 10.1172/jci.insight.89904 28138560 PMC5256142

[59] Cheng S Ma J Guo A Lu P Leonard JP Coleman M BTK inhibition targets in vivo CLL proliferation through its effects on B-cell receptor signaling activity. Leukemia (2014) 28:649–57. 10.1038/leu.2013.358 24270740

[60] Krysov S Steele AJ Coelho V Linley A Sanchez Hidalgo M Carter M Stimulation of surface IgM of chronic lymphocytic leukemia cells induces an unfolded protein response dependent on BTK and SYK. Blood (2014) 124:3101–9. 10.1182/blood-2014-04-567198 PMC4231419 25170122

[61] Ponader S Chen S-S Buggy JJ Balakrishnan K Gandhi V Wierda WG The Bruton tyrosine kinase inhibitor PCI-32765 thwarts chronic lymphocytic leukemia cell survival and tissue homing in vitro and in vivo. Blood (2012) 119:1182–9. 10.1182/blood-2011-10-386417 PMC4916557 22180443

[62] Chen S-S Chang BY Chang S Tong T Ham S Sherry B BTK inhibition results in impaired CXCR4 chemokine receptor surface expression, signaling and function in chronic lymphocytic leukemia. Leukemia (2016) 30:833–43. 10.1038/leu.2015.316 PMC4832074 26582643

[63] de Rooij MFM Kuil A Geest CR Eldering E Chang BY Buggy JJ The clinically active BTK inhibitor PCI-32765 targets B-cell receptor- and chemokine-controlled adhesion and migration in chronic lymphocytic leukemia. Blood (2012) 119:2590–4. 10.1182/blood-2011-11-390989 22279054

[64] Ortolano S Hwang I-Y Han S-B Kehrl JH Roles for phosphoinositide 3-kinases, Bruton’s tyrosine kinase, and Jun kinases in B lymphocyte chemotaxis and homing. Eur J Immunol (2006) 36:1285–95. 10.1002/eji.200535799 16619289

[65] Rendeiro AF Krausgruber T Fortelny N Zhao F Penz T Farlik M Chromatin mapping and single-cell immune profiling define the temporal dynamics of ibrutinib response in CLL. Nat Commun (2020) 11:577. 10.1038/s41467-019-14081-6 31996669 PMC6989523

[66] Guinn D Ruppert AS Maddocks K Jaglowski S Gordon A Lin TS miR-155 expression is associated with chemoimmunotherapy outcome and is modulated by Bruton’s tyrosine kinase inhibition with Ibrutinib. Leukemia (2015) 29:1210–3. 10.1038/leu.2014.344 PMC4424166 25486872

[67] Saleh LM Wang W Herman SEM Saba NS Anastas V Barber E Ibrutinib downregulates a subset of miRNA leading to upregulation of tumor suppressors and inhibition of cell proliferation in chronic lymphocytic leukemia. Leukemia (2017) 31:340–9. 10.1038/leu.2016.181 PMC9119706 27431016

[68] Furman RR Cheng S Lu P Setty M Perez AR Guo A Ibrutinib Resistance in Chronic Lymphocytic Leukemia. New Engl J Med (2014) 370:2352–4. 10.1056/NEJMc1402716 PMC4512173 24869597

[69] Chiron D Di Liberto M Martin P Huang X Sharman J Blecua P Cell-cycle reprogramming for PI3K inhibition overrides a relapse-specific C481S BTK mutation revealed by longitudinal functional genomics in mantle cell lymphoma. Cancer Discovery (2014) 4:1022–35. 10.1158/2159-8290.CD-14-0098 PMC4155003 25082755

[70] Epperla N Shana’ah AY Jones D Christian BA Ayyappan S Maddocks K Resistance mechanism for ibrutinib in marginal zone lymphoma. Blood Adv (2019) 3:500–2. 10.1182/bloodadvances.2018029058 PMC6391658 30760464

[71] Xu L Tsakmaklis N Yang G Chen JG Liu X Demos M Acquired mutations associated with ibrutinib resistance in Waldenström macroglobulinemia. Blood (2017) 129:2519–25. 10.1182/blood-2017-01-761726 PMC7484977 28235842

[72] Brandhuber B Gomez E Smith S Eary T Spencer S Rothenberg SM LOXO-305, A Next Generation Reversible BTK Inhibitor, for Overcoming Acquired Resistance to Irreversible BTK Inhibitors. Clin Lymphoma Myeloma Leukemia (2018) 18:S216. 10.1016/j.clml.2018.07.081

[73] Byrd JC Smith S Wagner-Johnston N Sharman J Chen AI Advani R First-in-human phase 1 study of the BTK inhibitor GDC-0853 in relapsed or refractory B-cell NHL and CLL. Oncotarget (2018) 9:13023–35. 10.18632/oncotarget.24310 PMC5849192 29560128

[74] Neuman LL Ward R Arnold D Combs DL Gruver D Hill W First-in-Human Phase 1a Study of the Safety, Pharmacokinetics, and Pharmacodynamics of the Noncovalent Bruton Tyrosine Kinase (BTK) Inhibitor SNS-062 in Healthy Subjects. Blood (2016) 128:2032–2. 10.1182/blood.V128.22.2032.2032

[75] Reiff SD Mantel R Smith LL Greene JT Muhowski EM Fabian CA The BTK Inhibitor ARQ 531 Targets Ibrutinib-Resistant CLL and Richter Transformation. Cancer Discovery (2018) 8:1300–15. 10.1158/2159-8290.CD-17-1409 PMC6261467 30093506

[76] Sun Y Ding N Song Y Yang Z Liu W Zhu J Degradation of Bruton’s tyrosine kinase mutants by PROTACs for potential treatment of ibrutinib-resistant non-Hodgkin lymphomas. Leukemia (2019) 33:2105–10. 10.1038/s41375-019-0440-x 30858551

[77] Sun Y Zhao X Ding N Gao H Wu Y Yang Y PROTAC-induced BTK degradation as a novel therapy for mutated BTK C481S induced ibrutinib-resistant B-cell malignancies. Cell Res (2018) 28:779–81. 10.1038/s41422-018-0055-1 PMC6028582 29875397

[78] Liu T-M Woyach JA Zhong Y Lozanski A Lozanski G Dong S Hypermorphic mutation of phospholipase C, γ2 acquired in ibrutinib-resistant CLL confers BTK independency upon B-cell receptor activation. Blood (2015) 126:61–8. 10.1182/blood-2015-02-626846 PMC4492196 25972157

[79] Walliser C Hermkes E Schade A Wiese S Deinzer J Zapatka M The Phospholipase Cγ2 Mutants R665W and L845F Identified in Ibrutinib-resistant Chronic Lymphocytic Leukemia Patients Are Hypersensitive to the Rho GTPase Rac2 Protein. J Biol Chem (2016) 291:22136–48. 10.1074/jbc.M116.746842 PMC5063995 27542411

[80] Kanagal-Shamanna R Jain P Patel KP Routbort M Bueso-Ramos C Alhalouli T Targeted multigene deep sequencing of Bruton tyrosine kinase inhibitor-resistant chronic lymphocytic leukemia with disease progression and Richter transformation. Cancer (2019) 125:559–74. 10.1002/cncr.31831 30508305

[81] Wu C de Miranda NF Chen L Wasik AM Mansouri L Jurczak W Genetic heterogeneity in primary and relapsed mantle cell lymphomas: Impact of recurrent CARD11 mutations. Oncotarget (2016) 7:38180–90. 10.18632/oncotarget.9500 PMC5122381 27224912

[82] Wilson WH Young RM Schmitz R Yang Y Pittaluga S Wright G Targeting B cell receptor signaling with ibrutinib in diffuse large B cell lymphoma. Nat Med (2015) 21:922–6. 10.1038/nm.3884 PMC8372245 26193343

[83] Xue L Apatira M Sirisawad M Chang B Abstract 1742: Ibrutinib plus proteasome or MALT1 inhibitors overcome resistance to BCR antagonists in CARD11 mutant-expressing B-lymphoma cells. In: Experimental and Molecular Therapeutics. Philadelphia, PA: American Association for Cancer Research (2015). p. 1742–2. 10.1158/1538-7445.AM2015-1742

[84] Lenz G Balasubramanian S Goldberg J Rizo A Schaffer M Phelps C Sequence variants in patients with primary and acquired resistance to ibrutinib in the phase 3 MCL3001 (RAY) trial. J Clin Oncol (2016) 34:7570–0. 10.1200/JCO.2016.34.15_suppl.7570

[85] Rahal R Frick M Romero R Korn JM Kridel R Chan FC Pharmacological and genomic profiling identifies NF-κB-targeted treatment strategies for mantle cell lymphoma. Nat Med (2014) 20:87–92. 10.1038/nm.3435 24362935

[86] Mohanty A Sandoval N Das M Pillai R Chen L Chen RW CCND1 mutations increase protein stability and promote ibrutinib resistance in mantle cell lymphoma. Oncotarget (2016) 7:73558–72. 10.18632/oncotarget.12434 PMC5341999 27713153

[87] Zhang L Yao Y Zhang S Liu Y Guo H Ahmed M Metabolic reprogramming toward oxidative phosphorylation identifies a therapeutic target for mantle cell lymphoma. Sci Transl Med (2019) 11(491):eaau1167. 10.1126/scitranslmed.aau1167 31068440

[88] Agarwal R Chan Y-C Tam CS Hunter T Vassiliadis D Teh CE Dynamic molecular monitoring reveals that SWI-SNF mutations mediate resistance to ibrutinib plus venetoclax in mantle cell lymphoma. Nat Med (2019) 25:119–29. 10.1038/s41591-018-0243-z 30455436

[89] Mondello P Brea EJ De Stanchina E Toska E Chang AY Fennell M Panobinostat acts synergistically with ibrutinib in diffuse large B cell lymphoma cells with MyD88 L265 mutations. JCI Insight (2017) 2:1–14. 10.1172/jci.insight.90196 PMC5358483 28352655

[90] Munshi M Liu X Chen JG Xu L Tsakmaklis N Demos MG SYK is activated by mutated MYD88 and drives pro-survival signaling in MYD88 driven B-cell lymphomas. Blood Cancer J (2020) 10:12. 10.1038/s41408-020-0277-6 32005797 PMC6994488

[91] Choi J Phelan JD Wright GW Häupl B Huang DW Shaffer AL Regulation of B cell receptor-dependent NF-κB signaling by the tumor suppressor KLHL14. Proc Natl Acad Sci USA (2020) 117:6092–102. 10.1073/pnas.1921187117 PMC7084139 32127472

[92] Cosson A Chapiro E Bougacha N Lambert J Herbi L Cung H-A Gain in the short arm of chromosome 2 (2p+) induces gene overexpression and drug resistance in chronic lymphocytic leukemia: analysis of the central role of XPO1. Leukemia (2017) 31:1625–9. 10.1038/leu.2017.100 28344316

[93] Burger JA Landau DA Taylor-Weiner A Bozic I Zhang H Sarosiek K Clonal evolution in patients with chronic lymphocytic leukaemia developing resistance to BTK inhibition. Nat Commun (2016) 7:11589. 10.1038/ncomms11589 27199251 PMC4876453

[94] Jiménez C Chan GG Xu L Tsakmaklis N Kofides A Demos MG Genomic evolution of ibrutinib-resistant clones in Waldenström macroglobulinaemia. Br J Haematol (2020) 189(6):1165–70. 10.1111/bjh.16463 PMC7299825 32103491

[95] Guerrera ML Tsakmaklis N Xu L Yang G Demos M Kofides A MYD88 mutated and wild-type Waldenström’s Macroglobulinemia: characterization of chromosome 6q gene losses and their mutual exclusivity with mutations in CXCR4. Haematologica (2018) 103:e408–11. 10.3324/haematol.2018.190181 PMC6119142 29599202

[96] Ahn IE Underbayev C Albitar A Herman SEM Tian X Maric I Clonal evolution leading to ibrutinib resistance in chronic lymphocytic leukemia. Blood (2017) 129:1469–79. 10.1182/blood-2016-06-719294 PMC5356450 28049639

[97] Quinquenel A Fornecker L-M Letestu R Ysebaert L Fleury C Lazarian G Prevalence of BTK and PLCG2 mutations in a real-life CLL cohort still on ibrutinib after 3 years: a FILO group study. Blood (2019) 134:641–4. 10.1182/blood.2019000854 31243043

[98] Scarfò L Bonfiglio S Sutton L-A Ljungström V Pandzic T Cortese D BTK and PLCG2 Mutations In Patients With Chronic Lymphocytic Leukemia Relapsing On Ibrutinib: A European Research Initiative On CLL (ERIC) Study Based On Real-World Evidence. In: 25th Congress of the European Hematology Association (2020). Abstract S161. Available at: https://library.ehaweb.org/eha/2020/eha25th/294981/lydia.scarf.btk.and.plcg2.mutations.in.patients.with.chronic.lymphocytic.html?f=menu%3D6%2Abrowseby%3D8%2Asortby%3D2%2Ace_id%3D1766%2Amarker%3D756.

[99] Gángó A Alpár D Galik B Marosvári D Kiss R Fésüs V Dissection of subclonal evolution by temporal mutation profiling in chronic lymphocytic leukemia patients treated with ibrutinib. Int J Cancer (2020) 146:85–93. 10.1002/ijc.32502 31180577

[100] Maddocks KJ Ruppert AS Lozanski G Heerema NA Zhao W Abruzzo L Etiology of Ibrutinib Therapy Discontinuation and Outcomes in Patients With Chronic Lymphocytic Leukemia. JAMA Oncol (2015) 1:80. 10.1001/jamaoncol.2014.218 26182309 PMC4520535

[101] Sharma S Galanina N Guo A Lee J Kadri S Van Slambrouck C Identification of a structurally novel BTK mutation that drives ibrutinib resistance in CLL. Oncotarget (2016) 7:68833–41. 10.18632/oncotarget.11932 PMC5356593 27626698

[102] Woyach JA Ruppert AS Guinn D Lehman A Blachly JS Lozanski A BTKC481S-Mediated Resistance to Ibrutinib in Chronic Lymphocytic Leukemia. J Clin Oncol (2017) 35:1437–43. 10.1200/JCO.2016.70.2282 PMC5455463 28418267

[103] Jones D Woyach JA Zhao W Caruthers S Tu H Coleman J PLCG2 C2 domain mutations co-occur with BTK and PLCG2 resistance mutations in chronic lymphocytic leukemia undergoing ibrutinib treatment. Leukemia (2017) 31:1645–7. 10.1038/leu.2017.110 28366935

[104] Guarini A Peragine N Messina M Marinelli M Ilari C Cafforio L Unravelling the suboptimal response of *TP53* -mutated chronic lymphocytic leukaemia to ibrutinib. Br J Haematol (2019) 184:392–6. 10.1111/bjh.15613 30338509

[105] Byrd JC Furman RR Coutre SE Burger JA Blum KA Coleman M Three-year follow-up of treatment-naïve and previously treated patients with CLL and SLL receiving single-agent ibrutinib. Blood (2015) 125:2497–506. 10.1182/blood-2014-10-606038 PMC4400288 25700432

[106] Brown JR Hillmen P O’Brien S Barrientos JC Reddy NM Coutre SE Extended follow-up and impact of high-risk prognostic factors from the phase 3 RESONATE study in patients with previously treated CLL/SLL. Leukemia (2018) 32:83–91. 10.1038/leu.2017.175 28592889 PMC5770586

[107] O’Brien S Furman RR Coutre S Flinn IW Burger JA Blum K Single-agent ibrutinib in treatment-naïve and relapsed/refractory chronic lymphocytic leukemia: a 5-year experience. Blood (2018) 131:1910–9. 10.1182/blood-2017-10-810044 PMC5921964 29437592

[108] Vallabhapurapu S Matsuzawa A Zhang W Tseng P-H Keats JJ Wang H Nonredundant and complementary functions of TRAF2 and TRAF3 in a ubiquitination cascade that activates NIK-dependent alternative NF-kappaB signaling. Nat Immunol (2008) 9:1364–70. 10.1038/ni.1678 PMC2671996 18997792

[109] Zarnegar BJ Wang Y Mahoney DJ Dempsey PW Cheung HH He J Noncanonical NF-kappaB activation requires coordinated assembly of a regulatory complex of the adaptors cIAP1, cIAP2, TRAF2 and TRAF3 and the kinase NIK. Nat Immunol (2008) 9:1371–8. 10.1038/ni.1676 PMC2676931 18997794

[110] Hunter ZR Xu L Yang G Zhou Y Liu X Cao Y The genomic landscape of Waldenstrom macroglobulinemia is characterized by highly recurring MYD88 and WHIM-like CXCR4 mutations, and small somatic deletions associated with B-cell lymphomagenesis. Blood (2014) 123:1637–46. 10.1182/blood-2013-09-525808 24366360

[111] Yang G Zhou Y Liu X Xu L Cao Y Manning RJ A mutation in MYD88 (L265P) supports the survival of lymphoplasmacytic cells by activation of Bruton tyrosine kinase in Waldenström macroglobulinemia. Blood (2013) 122:1222–32. 10.1182/blood-2012-12-475111 23836557

[112] Cao Y Hunter ZR Liu X Xu L Yang G Chen J The WHIM-like CXCR4S338X somatic mutation activates AKT and ERK, and promotes resistance to ibrutinib and other agents used in the treatment of Waldenstrom’s Macroglobulinemia. Leukemia (2015) 29:169–76. 10.1038/leu.2014.187 24912431

[113] Cao Y Hunter ZR Liu X Xu L Yang G Chen J CXCR4 WHIM-like frameshift and nonsense mutations promote ibrutinib resistance but do not supplant MYD88(L265P) -directed survival signalling in Waldenström macroglobulinaemia cells. Br J Haematol (2015) 168:701–7. 10.1111/bjh.13200 25371371

[114] Younes A Sehn LH Johnson P Zinzani PL Hong X Zhu J Randomized Phase III Trial of Ibrutinib and Rituximab Plus Cyclophosphamide, Doxorubicin, Vincristine, and Prednisone in Non-Germinal Center B-Cell Diffuse Large B-Cell Lymphoma. J Clin Oncol (2019) 37:1285–95. 10.1200/JCO.18.02403 PMC6553835 30901302

[115] Phelan JD Young RM Webster DE Roulland S Wright GW Kasbekar M A multiprotein supercomplex controlling oncogenic signalling in lymphoma. Nature (2018) 560:387–91. 10.1038/s41586-018-0290-0 PMC6201842 29925955

[116] Arora VK Schenkein E Murali R Subudhi SK Wongvipat J Balbas MD Glucocorticoid receptor confers resistance to antiandrogens by bypassing androgen receptor blockade. Cell (2013) 155:1309–22. 10.1016/j.cell.2013.11.012 PMC3932525 24315100

[117] Moriceau G Hugo W Hong A Shi H Kong X Yu CC Tunable-combinatorial mechanisms of acquired resistance limit the efficacy of BRAF/MEK cotargeting but result in melanoma drug addiction. Cancer Cell (2015) 27:240–56. 10.1016/j.ccell.2014.11.018 PMC4326539 25600339

[118] Muranen T Selfors LM Worster DT Iwanicki MP Song L Morales FC Inhibition of PI3K/mTOR leads to adaptive resistance in matrix-attached cancer cells. Cancer Cell (2012) 21:227–39. 10.1016/j.ccr.2011.12.024 PMC3297962 22340595

[119] Nazarian R Shi H Wang Q Kong X Koya RC Lee H Melanomas acquire resistance to B-RAF(V600E) inhibition by RTK or N-RAS upregulation. Nature (2010) 468:973–7. 10.1038/nature09626 PMC3143360 21107323

[120] Wilson FH Johannessen CM Piccioni F Tamayo P Kim JW Van Allen EM A functional landscape of resistance to ALK inhibition in lung cancer. Cancer Cell (2015) 27:397–408. 10.1016/j.ccell.2015.02.005 25759024 PMC4398996

[121] Kapoor I Li Y Sharma A Zhu H Bodo J Xu W Resistance to BTK inhibition by ibrutinib can be overcome by preventing FOXO3a nuclear export and PI3K/AKT activation in B-cell lymphoid malignancies. Cell Death Dis (2019) 10:924. 10.1038/s41419-019-2158-0 31801949 PMC6892912

[122] Zhang L Nomie K Zhang H Bell T Pham L Kadri S B-Cell Lymphoma Patient-Derived Xenograft Models Enable Drug Discovery and Are a Platform for Personalized Therapy. Clin Cancer Res (2017) 23:4212–23. 10.1158/1078-0432.CCR-16-2703 PMC5540787 28348046

[123] Zhao X Lwin T Silva A Shah B Tao J Fang B Unification of de novo and acquired ibrutinib resistance in mantle cell lymphoma. Nat Commun (2017) 8:14920. 10.1038/ncomms14920 28416797 PMC5399304

[124] Guan J Huang D Yakimchuk K Okret S p110α Inhibition Overcomes Stromal Cell–Mediated Ibrutinib Resistance in Mantle Cell Lymphoma. Mol Cancer Ther (2018) 17:1090–100. 10.1158/1535-7163.MCT-17-0784 29483220

[125] Lukas M Velten B Sellner L Tomska K Hüellein J Walther T Survey of ex vivo drug combination effects in chronic lymphocytic leukemia reveals synergistic drug effects and genetic dependencies. Leukemia (2020). 10.1038/s41375-020-0846-5 PMC7584477 32404973

[126] de Rooij MFM Kuil A Kater AP Kersten MJ Pals ST Spaargaren M Ibrutinib and idelalisib synergistically target BCR-controlled adhesion in MCL and CLL: a rationale for combination therapy. Blood (2015) 125:2306–9. 10.1182/blood-2014-12-619163 PMC4416940 25838279

[127] Davids MS Kim HT Nicotra A Savell A Francoeur K Hellman JM Umbralisib in combination with ibrutinib in patients with relapsed or refractory chronic lymphocytic leukaemia or mantle cell lymphoma: a multicentre phase 1-1b study. Lancet Haematol (2019) 6:e38–47. 10.1016/S2352-3026(18)30196-0 PMC7132223 30558987

[128] Schmidl C Vladimer GI Rendeiro AF Schnabl S Krausgruber T Taubert C Combined chemosensitivity and chromatin profiling prioritizes drug combinations in CLL. Nat Chem Biol (2019) 15:232–40. 10.1038/s41589-018-0205-2 PMC6746620 30692684

[129] Schaffer M Chaturvedi S Davis C Aquino R Stepanchick E Versele M Identification of potential ibrutinib combinations in hematological malignancies using a combination high-throughput screen. Leuk Lymphoma (2018) 59:931–40. 10.1080/10428194.2017.1349899 28750570

[130] Hing ZA Fung HYJ Ranganathan P Mitchell S El-Gamal D Woyach JA Next-generation XPO1 inhibitor shows improved efficacy and in vivo tolerability in hematological malignancies. Leukemia (2016) 30:2364–72. 10.1038/leu.2016.136 PMC5143172 27323910

[131] Hing ZA Mantel R Beckwith KA Guinn D Williams E Smith LL Selinexor is effective in acquired resistance to ibrutinib and synergizes with ibrutinib in chronic lymphocytic leukemia. Blood (2015) 125:3128–32. 10.1182/blood-2015-01-621391 PMC4432007 25838351

[132] Ming M Wu W Xie B Sukhanova M Wang W Kadri S XPO1 Inhibitor Selinexor Overcomes Intrinsic Ibrutinib Resistance in Mantle Cell Lymphoma via Nuclear Retention of IκB. Mol Cancer Ther (2018) 17:2564–74. 10.1158/1535-7163.MCT-17-0789-ATR 30510142

[133] Guo A Lu P Coffey G Conley P Pandey A Wang YL Dual SYK/JAK inhibition overcomes ibrutinib resistance in chronic lymphocytic leukemia: Cerdulatinib, but not ibrutinib, induces apoptosis of tumor cells protected by the microenvironment. Oncotarget (2017) 8:12953–67. 10.18632/oncotarget.14588 PMC5355069 28088788

[134] Lee J Zhang LL Wu W Guo H Li Y Sukhanova M Activation of MYC, a bona fide client of HSP90, contributes to intrinsic ibrutinib resistance in mantle cell lymphoma. Blood Adv (2018) 2:2039–51. 10.1182/bloodadvances.2018016048 PMC6113611 30115641

[135] Forestieri G Terzi di Bergamo L Loh JW Spina V Zucchetto A Condoluci A Mechanisms Of Adaptation To Ibrutinib In High Risk Chronic Lymphocytic Leukemia. In: 25th Congress of the European Hematology Association (2020). Abstract S154. Available at: https://library.ehaweb.org/eha/2020/eha25th/294974/gabriela.forestieri.mechanisms.of.adaptation.to.ibrutinib.in.high.risk.chronic.html?f=listing%3D4%2Abrowseby%3D8%2Asortby%3D2%2Amedia%3D3%2Aspeaker%3D663813.

[136] Gaudio E Tarantelli C Kwee I Barassi C Bernasconi E Rinaldi A Combination of the MEK inhibitor pimasertib with BTK or PI3K-delta inhibitors is active in preclinical models of aggressive lymphomas. Ann Oncol (2016) 27:1123–8. 10.1093/annonc/mdw131 26961147

[137] Hillmen P Rawstron AC Brock K Muñoz-Vicente S Yates FJ Bishop R Ibrutinib Plus Venetoclax in Relapsed/Refractory Chronic Lymphocytic Leukemia: The CLARITY Study. J Clin Oncol (2019) 37:2722–9. 10.1200/JCO.19.00894 PMC6879312 31295041

[138] Jain N Keating M Thompson P Ferrajoli A Burger J Borthakur G Ibrutinib and Venetoclax for First-Line Treatment of CLL. N Engl J Med (2019) 380:2095–103. 10.1056/NEJMoa1900574 PMC11827445 31141631

[139] Cervantes-Gomez F Lamothe B Woyach JA Wierda WG Keating MJ Balakrishnan K Pharmacological and Protein Profiling Suggests Venetoclax (ABT-199) as Optimal Partner with Ibrutinib in Chronic Lymphocytic Leukemia. Clin Cancer Res (2015) 21:3705–15. 10.1158/1078-0432.CCR-14-2809 PMC4537801 25829398

[140] Kater AP Seymour JF Hillmen P Eichhorst B Langerak AW Owen C Fixed Duration of Venetoclax-Rituximab in Relapsed/Refractory Chronic Lymphocytic Leukemia Eradicates Minimal Residual Disease and Prolongs Survival: Post-Treatment Follow-Up of the MURANO Phase III Study. J Clin Oncol (2019) 37:269–77. 10.1200/JCO.18.01580 30523712

[141] Tahir SK Smith ML Hessler P Rapp LR Idler KB Park CH Potential mechanisms of resistance to venetoclax and strategies to circumvent it. BMC Cancer (2017) 17:399. 10.1186/s12885-017-3383-5 28578655 PMC5457565

[142] Kuo H-P Ezell SA Schweighofer KJ Cheung LWK Hsieh S Apatira M Combination of Ibrutinib and ABT-199 in Diffuse Large B-Cell Lymphoma and Follicular Lymphoma. Mol Cancer Ther (2017) 16:1246–56. 10.1158/1535-7163.MCT-16-0555 28428442

[143] Wang YLL Franzen C Wang S Venkataraman G Li L Niu N Ibrutinib and Venetoclax Target Distinct Subpopulation of CLL Cells: Rationale for Drug Combination and Implication of Minimal Residual Disease Eradication. Blood (2019) 134:475–5. 10.1182/blood-2019-125396

[144] Galicia-Vázquez G Aloyz R Ibrutinib Resistance Is Reduced by an Inhibitor of Fatty Acid Oxidation in Primary CLL Lymphocytes. Front Oncol (2018) 8:411. 10.3389/fonc.2018.00411 30319974 PMC6168640

[145] Mraz M Zent CS Church AK Jelinek DF Wu X Pospisilova S Bone marrow stromal cells protect lymphoma B-cells from rituximab-induced apoptosis and targeting integrin α-4-β-1 (VLA-4) with natalizumab can overcome this resistance. Br J Haematol (2011) 155:53–64. 10.1111/j.1365-2141.2011.08794.x 21749361 PMC4405035

[146] Mathur R Sehgal L Braun FK Berkova Z Romaguerra J Wang M Targeting Wnt pathway in mantle cell lymphoma-initiating cells. J Hematol Oncol (2015) 8:63. 10.1186/s13045-015-0161-1 26048374 PMC4460883

[147] Chen JG Liu X Munshi M Xu L Tsakmaklis N Demos MG BTKCys481Ser drives ibrutinib resistance via ERK1/2 and protects BTKwild-type MYD88-mutated cells by a paracrine mechanism. Blood (2018) 131:2047–59. 10.1182/blood-2017-10-811752 29496671

[148] Rauert-Wunderlich H Rudelius M Berberich I Rosenwald A CD40L mediated alternative NFκB-signaling induces resistance to BCR-inhibitors in patients with mantle cell lymphoma. Cell Death Dis (2018) 9:86. 10.1038/s41419-017-0157-6 29367645 PMC5833745

[149] Sun Z Luo L Abstract 1298: CD40L-CD40 signaling on B-cell lymphoma response to BTK inhibitors. In: Experimental and Molecular Therapeutics. New Orleans, LA: American Association for Cancer Research (2016). p. 1298–8. 10.1158/1538-7445.AM2016-1298

[150] Lam K-P Kühn R Rajewsky K In Vivo Ablation of Surface Immunoglobulin on Mature B Cells by Inducible Gene Targeting Results in Rapid Cell Death. Cell (1997) 90:1073–83. 10.1016/S0092-8674(00)80373-6 9323135

[151] Srinivasan L Sasaki Y Calado DP Zhang B Paik JH DePinho RA PI3 kinase signals BCR-dependent mature B cell survival. Cell (2009) 139:573–86. 10.1016/j.cell.2009.08.041 PMC2787092 19879843

[152] Kim JH Kim WS Ryu K Kim SJ Park C CD79B limits response of diffuse large B cell lymphoma to ibrutinib. Leukemia Lymphoma (2016) 57:1413–22. 10.3109/10428194.2015.1113276 26699656

[153] Ma J Lu P Guo A Cheng S Zong H Martin P Characterization of ibrutinib-sensitive and -resistant mantle lymphoma cells. Br J Haematol (2014) 166:849–61. 10.1111/bjh.12974 24957109

[154] Herman SEM Mustafa RZ Jones J Wong DH Farooqui M Wiestner A Treatment with Ibrutinib Inhibits BTK- and VLA-4-Dependent Adhesion of Chronic Lymphocytic Leukemia Cells In Vivo. Clin Cancer Res (2015) 21:4642–51. 10.1158/1078-0432.CCR-15-0781 PMC4609275 26089373

[155] Chen L Ouyang J Wienand K Bojarczuk K Hao Y Chapuy B CXCR4 upregulation is an indicator of sensitivity to B-cell receptor/PI3K blockade and a potential resistance mechanism in B-cell receptor-dependent diffuse large B-cell lymphomas. Haematologica (2020) 105:1361–8. 10.3324/haematol.2019.216218 PMC7193488 31471373

[156] Chiodin G Dutton D Martino EA Drennan S Tracy I Ondrisova L High Surface IgM Levels Associate with Shorter Response Duration and Bypass of the BTK Blockade during Ibrutinib Therapy in CLL Patients. Blood (2019) 134:1752–2. 10.1182/blood-2019-128899

[157] Drennan S Chiodin G D’Avola A Tracy I Johnson PW Trentin L Ibrutinib Therapy Releases Leukemic Surface IgM from Antigen Drive in Chronic Lymphocytic Leukemia Patients. Clin Cancer Res (2019) 25:2503–12. 10.1158/1078-0432.CCR-18-1286 30373751

[158] Filip D Mraz M The role of MYC in the transformation and aggressiveness of “indolent” B-cell malignancies. Leuk Lymphoma (2020) 61:510–24. 10.1080/10428194.2019.1675877 31631728

[159] Varano G Raffel S Sormani M Zanardi F Lonardi S Zasada C The B-cell receptor controls fitness of MYC-driven lymphoma cells via GSK3β inhibition. Nature (2017) 546:302–6. 10.1038/nature22353 28562582

[160] Boissard F Fournié J-J Quillet-Mary A Ysebaert L Poupot M Nurse-like cells mediate ibrutinib resistance in chronic lymphocytic leukemia patients. Blood Cancer J (2015) 5:e355–5. 10.1038/bcj.2015.74 PMC4635187 26430726

[161] Jayappa KD Portell CA Gordon VL Capaldo BJ Bekiranov S Axelrod MJ Microenvironmental agonists generate de novo phenotypic resistance to combined ibrutinib plus venetoclax in CLL and MCL. Blood Adv (2017) 1:933–46. 10.1182/bloodadvances.2016004176 PMC5637393 29034364

[162] Chen S-S Tam CS Ramsay AG Ravichandran P Couto-Francisco NC Ibrahim M CLL B Cells Develop Resistance to Ibrutinib By Reinvigorating the IL-4R - IL-4 Axis Blocked By Bruton’s Tyrosine Kinase Inhibitors Including Acalabrutinib and Zanubrutinib. Blood (2019) 134:477–7. 10.1182/blood-2019-127255

[163] Chen Z Ayala P Wang M Fayad L Katz RL Romaguera J Prospective isolation of clonogenic mantle cell lymphoma-initiating cells. Stem Cell Res (2010) 5:212–25. 10.1016/j.scr.2010.07.003 PMC2952723 20851072

[164] Jung HJ Chen Z McCarty N Stem-like tumor cells confer drug resistant properties to mantle cell lymphoma. Leuk Lymphoma (2011) 52:1066–79. 10.3109/10428194.2011.562570 21599592

[165] Lee C-G Das B Lin TL Grimes C Zhang X Lavezzi T A rare fraction of drug-resistant follicular lymphoma cancer stem cells interacts with follicular dendritic cells to maintain tumourigenic potential. Br J Haematol (2012) 158:79–90. 10.1111/j.1365-2141.2012.09123.x 22509798 PMC3374069

[166] Medina DJ Abass-Shereef J Walton K Goodell L Aviv H Strair RK Cobblestone-area forming cells derived from patients with mantle cell lymphoma are enriched for CD133+ tumor-initiating cells. PloS One (2014) 9:e91042. 10.1371/journal.pone.0091042 24722054 PMC3982953

[167] O’Brien SM Furman RR Coutre SE Flinn IW Burger J Blum K Five-Year Experience with Single-Agent Ibrutinib in Patients with Previously Untreated and Relapsed/Refractory Chronic Lymphocytic Leukemia/Small Lymphocytic Leukemia. Blood (2016) 128:233–3. 10.1182/blood.V128.22.233.233

[168] Awan FT Schuh A Brown JR Furman RR Pagel JM Hillmen P Acalabrutinib monotherapy in patients with chronic lymphocytic leukemia who are intolerant to ibrutinib. Blood Adv (2019) 3:1553–62. 10.1182/bloodadvances.2018030007 PMC6517672 31088809

[169] Barf T Covey T Izumi R van de Kar B Gulrajani M van Lith B Acalabrutinib (ACP-196): A Covalent Bruton Tyrosine Kinase Inhibitor with a Differentiated Selectivity and In Vivo Potency Profile. J Pharmacol Exp Ther (2017) 363:240–52. 10.1124/jpet.117.242909 28882879

[170] Sharman JP Egyed M Jurczak W Skarbnik A Pagel JM Flinn IW Acalabrutinib with or without obinutuzumab versus chlorambucil and obinutuzmab for treatment-naive chronic lymphocytic leukaemia (ELEVATE TN): a randomised, controlled, phase 3 trial. Lancet (2020) 395:1278–91. 10.1016/S0140-6736(20)30262-2 PMC8151619 32305093

[171] Tam CS Trotman J Opat S Burger JA Cull G Gottlieb D Phase 1 study of the selective BTK inhibitor zanubrutinib in B-cell malignancies and safety and efficacy evaluation in CLL. Blood (2019) 134:851–9. 10.1182/blood.2019001160 PMC6742923 31340982

[172] Wang M Rule S Zinzani PL Goy A Casasnovas O Smith SD Durable response with single-agent acalabrutinib in patients with relapsed or refractory mantle cell lymphoma. Leukemia (2019) 33:2762–6. 10.1038/s41375-019-0575-9 31558766

[173] George B Mullick Chowdhury S Hart A Sircar A Singh SK Nath UK Ibrutinib Resistance Mechanisms and Treatment Strategies for B-Cell Lymphomas. Cancers (2020) 12:1328. 10.3390/cancers12051328 PMC7281539 32455989

[174] Thijssen R Ter Burg J Garrick B van Bochove GGW Brown JR Fernandes SM Dual TORK/DNA-PK inhibition blocks critical signaling pathways in chronic lymphocytic leukemia. Blood (2016) 128:574–83. 10.1182/blood-2016-02-700328 27235137

[175] Delvecchio VS Sana I Mantione ME Vilia MG Ranghetti P Rovida A Interleukin-1 receptor-associated kinase 4 inhibitor interrupts toll-like receptor signalling and sensitizes chronic lymphocytic leukaemia cells to apoptosis. Br J Haematol (2020) 189:475–88. 10.1111/bjh.16386 32057093

[176] Ngo VN Young RM Schmitz R Jhavar S Xiao W Lim K-H Oncogenically active MYD88 mutations in human lymphoma. Nature (2011) 470:115–9. 10.1038/nature09671 PMC5024568 21179087

[177] Roberts AW Ma S Kipps TJ Coutre SE Davids MS Eichhorst B Efficacy of venetoclax in relapsed chronic lymphocytic leukemia is influenced by disease and response variables. Blood (2019) 134:111–22. 10.1182/blood.2018882555 PMC6624969 31023700

[178] Goy A Ramchandren R Ghosh N Munoz J Morgan DS Dang NH Ibrutinib plus lenalidomide and rituximab has promising activity in relapsed/refractory non–germinal center B-cell–like DLBCL. Blood (2019) 134:1024–36. 10.1182/blood.2018891598 PMC6764267 31331917

[179] Jerkeman M Eskelund CW Hutchings M Räty R Wader KF Laurell A Ibrutinib, lenalidomide, and rituximab in relapsed or refractory mantle cell lymphoma (PHILEMON): a multicentre, open-label, single-arm, phase 2 trial. Lancet Haematol (2018) 5:e109–16. 10.1016/S2352-3026(18)30018-8 29396091

[180] Ujjani C Wang H Skarbnik A Trivedi N Ramzi P Khan N A phase 1 study of lenalidomide and ibrutinib in combination with rituximab in relapsed and refractory CLL. Blood Adv (2018) 2:762–8. 10.1182/bloodadvances.2017015263 PMC5894261 29610115

[181] Yang Y Shaffer AL Emre NCT Ceribelli M Zhang M Wright G Exploiting Synthetic Lethality for the Therapy of ABC Diffuse Large B Cell Lymphoma. Cancer Cell (2012) 21:723–37. 10.1016/j.ccr.2012.05.024 PMC4059833 22698399

[182] Burger JA Sivina M Jain N Kim E Kadia T Estrov Z Randomized trial of ibrutinib vs ibrutinib plus rituximab in patients with chronic lymphocytic leukemia. Blood (2019) 133:1011–9. 10.1182/blood-2018-10-879429 PMC6405333 30530801

[183] Skarzynski M Niemann CU Lee YS Martyr S Maric I Salem D Interactions between Ibrutinib and Anti-CD20 Antibodies: Competing Effects on the Outcome of Combination Therapy. Clin Cancer Res (2016) 22:86–95. 10.1158/1078-0432.CCR-15-1304 26283682 PMC4703510

[184] Albertsson-Lindblad A Freiburghaus C Jerkeman M Ek S Ibrutinib inhibits antibody dependent cellular cytotoxicity induced by rituximab or obinutuzumab in MCL cell lines, not overcome by addition of lenalidomide. Exp Hematol Oncol (2019) 8:16. 10.1186/s40164-019-0141-1 31406628 PMC6685275

[185] Moreno C Greil R Demirkan F Tedeschi A Anz B Larratt L Ibrutinib plus obinutuzumab versus chlorambucil plus obinutuzumab in first-line treatment of chronic lymphocytic leukaemia (iLLUMINATE): a multicentre, randomised, open-label, phase 3 trial. Lancet Oncol (2019) 20:43–56. 10.1016/S1470-2045(18)30788-5 30522969

[186] Buchner M Brantner P Stickel N Prinz G Burger M Bär C The microenvironment differentially impairs passive and active immunotherapy in chronic lymphocytic leukaemia - CXCR4 antagonists as potential adjuvants for monoclonal antibodies. Br J Haematol (2010) 151:167–78. 10.1111/j.1365-2141.2010.08316.x 20738306

[187] Choi MY Widhopf GF Ghia EM Kidwell RL Hasan MK Yu J Phase I Trial: Cirmtuzumab Inhibits ROR1 Signaling and Stemness Signatures in Patients with Chronic Lymphocytic Leukemia. Cell Stem Cell (2018) 22:951–9.e3. 10.1016/j.stem.2018.05.018 29859176 PMC7001723

[188] Fischer K Al-Sawaf O Bahlo J Fink A-M Tandon M Dixon M Venetoclax and Obinutuzumab in Patients with CLL and Coexisting Conditions. New Engl J Med (2019) 380:2225–36. 10.1056/NEJMoa1815281 31166681

[189] Mato AR Hill BT Lamanna N Barr PM Ujjani CS Brander DM Optimal sequencing of ibrutinib, idelalisib, and venetoclax in chronic lymphocytic leukemia: results from a multicenter study of 683 patients. Ann Oncol (2017) 28:1050–6. 10.1093/annonc/mdx031 28453705

[190] Dai B Grau M Juilland M Klener P Höring E Molinsky J B-cell receptor-driven MALT1 activity regulates MYC signaling in mantle cell lymphoma. Blood (2017) 129:333–46. 10.1182/blood-2016-05-718775 27864294

[191] El-Gamal D Williams K LaFollette TD Cannon M Blachly JS Zhong Y PKC-β as a therapeutic target in CLL: PKC inhibitor AEB071 demonstrates preclinical activity in CLL. Blood (2014) 124:1481–91. 10.1182/blood-2014-05-574830 PMC4148770 25001469

[192] Fontán L Qiao Q Hatcher JM Casalena G Us I Teater M Specific covalent inhibition of MALT1 paracaspase suppresses B cell lymphoma growth. J Clin Invest (2018) 128:4397–412. 10.1172/JCI99436 PMC6159983 30024860

[193] Saba NS Wong DH Tanios G Iyer JR Lobelle-Rich P Dadashian EL MALT1 Inhibition Is Efficacious in Both Naïve and Ibrutinib-Resistant Chronic Lymphocytic Leukemia. Cancer Res (2017) 77:7038–48. 10.1158/0008-5472.CAN-17-2485 PMC5732856 28993409

[194] Gobessi S Laurenti L Longo PG Carsetti L Berno V Sica S Inhibition of constitutive and BCR-induced Syk activation downregulates Mcl-1 and induces apoptosis in chronic lymphocytic leukemia B cells. Leukemia (2009) 23:686–97. 10.1038/leu.2008.346 19092849

[195] Zhuang J Hawkins SF Glenn MA Lin K Johnson GG Carter A Akt is activated in chronic lymphocytic leukemia cells and delivers a pro-survival signal: the therapeutic potential of Akt inhibition. Haematologica (2010) 95:110–8. 10.3324/haematol.2009.010272 PMC2805750 19713228

[196] Ramadani F Bolland DJ Garcon F Emery JL Vanhaesebroeck B Corcoran AE The PI3K Isoforms p110 and p110 Are Essential for Pre-B Cell Receptor Signaling and B Cell Development. Sci Signaling (2010) 3:ra60–0. 10.1126/scisignal.2001104 PMC3540743 20699475

[197] Ali AY Wu X Eissa N Hou S Ghia J-E Murooka TT Distinct roles for phosphoinositide 3-kinases γ and δ in malignant B cell migration. Leukemia (2018) 32:1958–69. 10.1038/s41375-018-0012-5 PMC6127087 29479062

[198] Hoellenriegel J Meadows SA Sivina M Wierda WG Kantarjian H Keating MJ The phosphoinositide 3′-kinase delta inhibitor, CAL-101, inhibits B-cell receptor signaling and chemokine networks in chronic lymphocytic leukemia. Blood (2011) 118:3603–12. 10.1182/blood-2011-05-352492 PMC4916562 21803855

[199] Lannutti BJ Meadows SA Herman SEM Kashishian A Steiner B Johnson AJ CAL-101, a p110delta selective phosphatidylinositol-3-kinase inhibitor for the treatment of B-cell malignancies, inhibits PI3K signaling and cellular viability. Blood (2011) 117:591–4. 10.1182/blood-2010-03-275305 PMC3694505 20959606

[200] Lampson BL Brown JR PI3Kδ-selective and PI3Kα/δ-combinatorial inhibitors in clinical development for B-cell non-Hodgkin lymphoma. Expert Opin Invest Drugs (2017) 26:1267–79. 10.1080/13543784.2017.1384815 PMC5747968 28945111

[201] Zelenetz AD Barrientos JC Brown JR Coiffier B Delgado J Egyed M Idelalisib or placebo in combination with bendamustine and rituximab in patients with relapsed or refractory chronic lymphocytic leukaemia: interim results from a phase 3, randomised, double-blind, placebo-controlled trial. Lancet Oncol (2017) 18:297–311. 10.1016/S1470-2045(16)30671-4 28139405 PMC5589180

[202] Liu N Rowley BR Bull CO Schneider C Haegebarth A Schatz CA BAY 80-6946 is a highly selective intravenous PI3K inhibitor with potent p110α and p110δ activities in tumor cell lines and xenograft models. Mol Cancer Ther (2013) 12:2319–30. 10.1158/1535-7163.MCT-12-0993-T 24170767

[203] de Frias M Iglesias-Serret D Cosialls AM González-Gironès DM Pérez-Perarnau A Rubio-Patiño C Isoform-selective phosphoinositide 3-kinase inhibitors induce apoptosis in chronic lymphocytic leukaemia cells. Br J Haematol (2010) 150:108–11. 10.1111/j.1365-2141.2010.08151.x 20230409

[204] Krause G Hassenrück F Hallek M Copanlisib for treatment of B-cell malignancies: the development of a PI3K inhibitor with considerable differences to idelalisib. DDDT (2018) 12:2577–90. 10.2147/DDDT.S142406 PMC6109662 30174412

[205] Mensah F Blaize J-P Bryan L Spotlight on copanlisib and its potential in the treatment of relapsed/refractory follicular lymphoma: evidence to date. OTT (2018) 11:4817–27. 10.2147/OTT.S142264 PMC6097514 30147333

[206] Furman RR Sharman JP Coutre SE Cheson BD Pagel JM Hillmen P Idelalisib and rituximab in relapsed chronic lymphocytic leukemia. N Engl J Med (2014) 370:997–1007. 10.1056/NEJMoa1315226 24450857 PMC4161365

[207] Ghia P Ljungström V Tausch E Agathangelidis A Scheffold A Scarfo L Whole-Exome Sequencing Revealed No Recurrent Mutations within the PI3K Pathway in Relapsed Chronic Lymphocytic Leukemia Patients Progressing Under Idelalisib Treatment. Blood (2016) 128:2770–0. 10.1182/blood.V128.22.2770.2770

[208] Scheffold A Jebaraj BMC Tausch E Bloehdorn J Ghia P Yahiaoui A IGF1R as druggable target mediating PI3K-δ inhibitor resistance in a murine model of chronic lymphocytic leukemia. Blood (2019) 134:534–47. 10.1182/blood.2018881029 PMC8212352 31010847

[209] Murali I Kasar S McWilliams EM Itchaki G Tyekucheva S Livitz D Activating MAPK Pathway Mutations Mediate Primary Resistance to PI3K Inhibitors in Chronic Lymphocytic Leukemia (CLL). Blood (2018) 132:587–7. 10.1182/blood-2018-99-115304

[210] Kim JH Kim WS Park C Interleukin-6 mediates resistance to PI3K-pathway–targeted therapy in lymphoma. BMC Cancer (2019) 19:936. 10.1186/s12885-019-6057-7 31601188 PMC6785854

