# Supplementary material for: Genetic and Non-Genetic Mechanisms of Resistance to BCR Signaling Inhibitors in B Cell Malignancies
Source: Front Oncol. 2020 Oct 26;10:591577. doi: 10.3389/fonc.2020.591577 (PMC7116322; doi:10.3389/fonc.2020.591577)
Supplement: Supplementary file 1 [file DataSheet_1.pdf]

Supplemental Materials

GENETIC AND NON-GENETIC MECHANISMS OF RESISTANCE TO BCR SIGNALING INHIBITORS IN B CELL MALIGNANCIES

Laura Ondrisova,[1,2] Marek Mraz[1,2*]

[1] Molecular Medicine, CEITEC Masaryk University, Brno, Czech Republic

[2] Department of Internal Medicine, Hematology and Oncology, University Hospital Brno and Faculty of Medicine, Masaryk University, Brno, Czech Republic.

*corresponding author

Correspondence:

Marek Mraz, M.D., Ph.D.
Associate Professor of Oncology
Central European Institute of Technology, Masaryk University
Kamenice 5, 625 00 Brno, Czech Republic
E-mail: marek.mraz@email.cz
Tel.: +420 549498143

Supplemental Tables: 2

Supplemental Table 1. IC50 values (nM) for BTK and off-target kinases for various BTK inhibitors

	Irreversible, covalent BTK inhibitors (Ref.)						Reversible, non-covalent BTK inhibitors (Ref.)					
	Ibrutinib (PCI-32765) (1)	Acalabrutinib (ACP-196) (1)	Tirabrutinib (ONO/GS-4059) (1)	Spebrutinib (CC-292) (1)	Zanubrutinib (BGB-3111) (2)	Zanubrutinib# (% inhibition) (3)	BMS-986142 (1)	Fenebrutinib (GDC-0853) (1)	LOXO-305 (4)	Vecabrutinib (SNS-062) (5)	ARQ 531 (6)	ARQ 531# (% inhibition) (6)
BTK	0.55	19.2	19.5	7.9	0.22	98%	1.5	2.3	3.15	3	0.85	98%
BLK	0.495	>1000	>1000	>1000	-	99%	110	>1000	4100	23	9.7	98%
BMX	1.83	425	34.6	7.53	-	98%	138	351	1155	224	5.2	101%
BRK	16.5	>1000	>1000	>1000	-	99%	>1000	>1000	54.3	-	2.5	100%
CSK	39.5	>1000	>1000	>1000	-	66%	353	>1000	-	-	46	92%
EGFR	23.2	>1000	>1000	>1000	606*	86%	>1000	>1000	>1000	6644	-	80%
EPHA1	>1000	>1000	>1000	>1000	-	5.5%	588	>1000	-	-	-	36%
EPHA7	>1000	>1000	>1000	>1000	-	-4%	>1000	>1000	-	-	-	7%
EPHB1	>1000	>1000	>1000	>1000	-	2.7%	>1000	>1000	-	-	-	10%
ERBB2	32.7	>1000	>1000	>1000	661	40%	>1000	>1000	-	-	-	-
ERBB4	2.7	78.2	>1000	>1000	-	96%	>1000	>1000	13.3	317	-	96%
FGR	2.63	>1000	>1000	>1000	-	76%	105	387	-	-	26	94%
FLT3	264	>1000	>1000	>1000	-	61%	>1000	>1000	-	-	-	36%
FLT4	-	-	-	-	-	-	-	-	-	-	-	99%
Fms	-	-	-	-	-	-	-	-	-	-	-	95%
FRK	44.1	>1000	>1000	>1000	-	71%	>1000	>1000	-	-	48	93%
FYN	-	-	-	-	-	50%	-	-	-	-	32	95%
HCK	27.6	>1000	>1000	>1000	-	65%	525	>1000	-	276	18	98%
IGF1R	>1000	>1000	>1000	>1000	-	-2.9%	756	>1000	-	-	-	39%
ITK	218	>1000	>1000	>1000	30	76%	>1000	>1000	>5000	14	>10,000	19%
JAK1	-	-	-	-	-	1.2%	-	-	>30000	-	-	-
JAK2	-	-	-	-	-	0.5%	-	-	ND	-	-	0%
JAK3	240	>1000	>1000	50.1	200	36%	>1000	>1000	ND	-	-	-16%
LCK	3.64	>1000	>1000	>1000	-	73%	149	>1000	-	8	3.9	94%
LYN	15.8	>1000	>1000	>1000	-	35%	>1000	>1000	-	-	19	100%
MEK1	-	-	-	-	-	57%	-	-	147	-	599	55%
MEK2	-	-	-	-	-	86%	-	-	82.7	-	-	-
MuSK	>1000	>1000	>1000	>1000	-	-2.8%	362	>1000	-	-	-	-
NEK11	-	-	-	-	-	19%	-	-	-	90	-	8%
NLK	-	-	-	-	-	29%	-	-	-	-	-	-3%
RAF1	-	-	-	-	-	-	-	-	-	-	35	77%
Ret	254	>1000	>1000	>1000	-	20%	682	>1000	-	-	-	98%
RIPK2	16	898	>1000	>1000	-	-8.7%	>1000	>1000	-	-	-	12%
Ros	>1000	>1000	>1000	>1000	-	-0.7%	>1000	>1000	-	-	-	-8%
SRC	26.1	>1000	>1000	>1000	-	29%	183	302	>5000	84	-	96%
Srm	5.78	>1000	>1000	>1000	-	31%	>1000	>1000	-	-	-	-
STK16	>1000	>1000	>1000	53.5	-	5.1%	>1000	>1000	-	-	-	-
TEC	10.2	>1000	240	9.51	1.9	79%	32.4	>1000	1234	14	5.8	90%
Tie2	-	-	-	-	-	3.3%	-	-	-	-	29	96%
TNK2	167	>1000	>1000	>1000	-	42%	>1000	>1000	-	-	-	90%
TrkA	>1000	>1000	>1000	>1000	-	-3.2%	155	>1000	-	-	13	95%
TrkB	>1000	>1000	>1000	>1000	-	-5.6%	429	>1000	-	-	12	99%
TrkC	-	-	-	-	-	3%	-	-	-	-	19	-
TXK	2.52	>1000	>1000	39.1	-	96%	147	>1000	209	474	36	93%
YES	4.37	>1000	>1000	>1000	-	51%	340	>1000	157	-	4.2	100%

" - " indicates unknown values

# data with "%" (grey columns) indicate percentage of inhibition after 1 uM inhibitor

* indicates values assessed in a cellular assay (other data indicate values from kinome assays with purified proteins)

Supplemental Table 2. List of the most common *BTK* and *PLCG2* mutations found in ibrutinib-resistant B cell malignancies.

Gene	Mutation	Disease	Mechanism of resistance	Reference
BTK	C481S	CLL, MCL, WM, MZL	causes reversible ibrutinib binding	(7–12)
	C481R/Y/F	CLL, WM, MCL	disrupts ibrutinib binding	(9,11–13)
	T474I/S	CLL	attenuates ibrutinib binding	(13,14)
	L528W	CLL	hinders ibrutinib binding	(13,14)
	R28S, G164D, R490H, Q516K	CLL	unknown	(15)
	T316A	CLL	unknown	(14)
PLCG2	R665W	CLL, MZL	BTK-independent activation by SYK and LYN	(7,10,11,16,17)
	L845F/V	CLL	BTK-independent activation	(7,11,13,17)
	S707Y/P/F	CLL	disrupts of an autoinhibitory SH2 domain	(7,11,13,17–19)
	P664S	CLL	disrupts of an autoinhibitory SH2 domain	(11)
	deletion of S707 and A708	CLL	disrupts of an autoinhibitory SH2 domain	(11)
	D933H/Y	CLL	mutation in catalytic domain	(15,17,19)
	Y495H	WM	unknown	(9)
	F82S, R694H, S1192G	CLL	unknown	(15)
	D334H, R742P, D1140G	CLL	unknown	(13)
	L484R	CLL	unknown	(20)
	deletion of E1139	CLL	unknown	(20)
	M1141R/K	CLL	unknown	(17,19)

Supplemental references

1. Crawford JJ, Johnson AR, Misner DL, Belmont LD, Castanedo G, Choy R, Coraggio M, Dong L, Eigenbrot C, Erickson R, et al. Discovery of GDC-0853: A Potent, Selective, and Noncovalent Bruton's Tyrosine Kinase Inhibitor in Early Clinical Development. *J Med Chem* (2018) **61**:2227–2245. doi:10.1021/acs.jmedchem.7b01712

2. Tam C, Grigg AP, Opat S, Ku M, Gilbertson M, Anderson MA, Seymour JF, Ritchie DS, Dicorleto C, Dimovski B, et al. The BTK Inhibitor, Bgb-3111, Is Safe, Tolerable, and Highly Active in Patients with Relapsed/ Refractory B-Cell Malignancies: Initial Report of a Phase 1 First-in-Human Trial. *Blood* (2015) **126**:832–832. doi:10.1182/blood.V126.23.832.832

3. Guo Y, Liu Y, Hu N, Yu D, Zhou C, Shi G, Zhang B, Wei M, Liu J, Luo L, et al. Discovery of Zanubrutinib (BGB-3111), a Novel, Potent, and Selective Covalent Inhibitor of Bruton's Tyrosine Kinase. *J Med Chem* (2019) **62**:7923–7940. doi:10.1021/acs.jmedchem.9b00687

4. Brandhuber B, Gomez E, Smith S, Eary T, Spencer S, Rothenberg SM, Andrews S. LOXO-305, A Next Generation Reversible BTK Inhibitor, for Overcoming Acquired Resistance to Irreversible BTK Inhibitors. *Clinical Lymphoma Myeloma and Leukemia* (2018) **18**:S216. doi:10.1016/j.clml.2018.07.081

5. Allan JN, Wierda WG, Patel K, O'Brien SM, Mato AR, Davids MS, Furman RR, Pagel JM, Fox JA, Ward R, et al. Preliminary Safety, Pharmacokinetic, and Pharmacodynamic Results from a Phase 1b/2 Dose-Escalation and Cohort-Expansion Study of the Noncovalent, Reversible Bruton's Tyrosine Kinase Inhibitor (BTKi), Vecabrutinib, in B-Lymphoid Malignancies. *Blood* (2018) **132**:3141–3141. doi:10.1182/blood-2018-99-116382

6. Reiff SD, Mantel R, Smith LL, Greene JT, Muhowski EM, Fabian CA, Goettl VM, Tran M, Harrington BK, Rogers KA, et al. The BTK Inhibitor ARQ 531 Targets Ibrutinib-Resistant CLL and Richter Transformation. *Cancer Discov* (2018) **8**:1300–1315. doi:10.1158/2159-8290.CD-17-1409

7. Woyach JA, Furman RR, Liu T-M, Ozer HG, Zapatka M, Ruppert AS, Xue L, Li DH-H, Steggerda SM, Versele M, et al. Resistance mechanisms for the Bruton's tyrosine kinase inhibitor ibrutinib. *N Engl J Med* (2014) **370**:2286–2294. doi:10.1056/NEJMoa1400029

8. Chiron D, Di Liberto M, Martin P, Huang X, Sharman J, Blecua P, Mathew S, Vijay P, Eng K, Ali S, et al. Cell-cycle reprogramming for PI3K inhibition overrides a relapse-specific C481S BTK mutation revealed by longitudinal functional genomics in mantle cell lymphoma. *Cancer Discov* (2014) **4**:1022–1035. doi:10.1158/2159-8290.CD-14-0098

9. Xu L, Tsakmaklis N, Yang G, Chen JG, Liu X, Demos M, Kofides A, Patterson CJ, Meid K, Gustine J, et al. Acquired mutations associated with ibrutinib resistance in Waldenström macroglobulinemia. *Blood* (2017) **129**:2519–2525. doi:10.1182/blood-2017-01-761726

10. Epperla N, Shana'ah AY, Jones D, Christian BA, Ayyappan S, Maddocks K, Woyach JA. Resistance mechanism for ibrutinib in marginal zone lymphoma. *Blood Advances* (2019) **3**:500–502. doi:10.1182/bloodadvances.2018029058

11. Ahn IE, Underbayev C, Albitar A, Herman SEM, Tian X, Maric I, Arthur DC, Wake L, Pittaluga S, Yuan CM, et al. Clonal evolution leading to ibrutinib resistance in chronic lymphocytic leukemia. *Blood* (2017) **129**:1469–1479. doi:10.1182/blood-2016-06-719294

12. Jain P, Kanagal-Shamanna R, Zhang S, Ahmed M, Ghorab A, Zhang L, Ok CY, Li S, Hagemeister F, Zeng D, et al. Long-term outcomes and mutation profiling of patients with mantle cell lymphoma (MCL) who discontinued ibrutinib. *Br J Haematol* (2018) **183**:578–587. doi:10.1111/bjh.15567

13. Maddocks KJ, Ruppert AS, Lozanski G, Heerema NA, Zhao W, Abruzzo L, Lozanski A, Davis M, Gordon A, Smith LL, et al. Etiology of Ibrutinib Therapy Discontinuation and Outcomes in Patients With Chronic Lymphocytic Leukemia. *JAMA Oncology* (2015) **1**:80. doi:10.1001/jamaoncol.2014.218

14. Sharma S, Galanina N, Guo A, Lee J, Kadri S, Van Slambrouck C, Long B, Wang W, Ming M, Furtado LV, et al. Identification of a structurally novel BTK mutation that drives ibrutinib resistance in CLL. *Oncotarget* (2016) **7**:68833–68841. doi:10.18632/oncotarget.11932

15. Gángó A, Alpár D, Galik B, Marosvári D, Kiss R, Fésüs V, Aczél D, Eyüpoglu E, Nagy N, Nagy Á, et al. Dissection of subclonal evolution by temporal mutation profiling in chronic lymphocytic leukemia patients treated with ibrutinib. *International Journal of Cancer* (2020) **146**:85–93. doi:10.1002/ijc.32502

16. Liu T-M, Woyach JA, Zhong Y, Lozanski A, Lozanski G, Dong S, Strattan E, Lehman A, Zhang X, Jones JA, et al. Hypermorphic mutation of phospholipase C, γ2 acquired in ibrutinib-resistant CLL confers BTK independency upon B-cell receptor activation. *Blood* (2015) **126**:61–68. doi:10.1182/blood-2015-02-626846

17. Jones D, Woyach JA, Zhao W, Caruthers S, Tu H, Coleman J, Byrd JC, Johnson AJ, Lozanski G. PLCG2 C2 domain mutations co-occur with BTK and PLCG2 resistance mutations in chronic lymphocytic leukemia undergoing ibrutinib treatment. *Leukemia* (2017) **31**:1645–1647. doi:10.1038/leu.2017.110

18. Zhou Q, Lee G-S, Brady J, Datta S, Katan M, Sheikh A, Martins MS, Bunney TD, Santich BH, Moir S, et al. A hypermorphic missense mutation in PLCG2, encoding phospholipase Cγ2, causes a dominantly inherited autoinflammatory disease with immunodeficiency. *Am J Hum Genet* (2012) **91**:713–720. doi:10.1016/j.ajhg.2012.08.006

19. Burger JA, Landau DA, Taylor-Weiner A, Bozic I, Zhang H, Sarosiek K, Wang L, Stewart C, Fan J, Hoellenriegel J, et al. Clonal evolution in patients with chronic lymphocytic leukaemia developing resistance to BTK inhibition. *Nat Commun* (2016) **7**:11589. doi:10.1038/ncomms11589

20. Landau DA, Sun C, Rosebrock D, Herman SEM, Fein J, Sivina M, Underbayev C, Liu D, Hoellenriegel J, Ravichandran S, et al. The evolutionary landscape of chronic lymphocytic leukemia treated with ibrutinib targeted therapy. *Nat Commun* (2017) **8**:2185. doi:10.1038/s41467-017-02329-y
